# A comprehensive numerical approach to coil placement in cerebral aneurysms: mathematical modeling and in silico occlusion classification

**DOI:** 10.1007/s10237-024-01882-y

**Published:** 2024-08-20

**Authors:** Fabian Holzberger, Markus Muhr, Barbara Wohlmuth

**Affiliations:** Department of Mathematics, Technical University of Munich, Boltzmannstr. 3/III, 85748 Garching b. München, Germany

**Keywords:** Discrete elastic rods, Cerebral aneurysm, Endovascular coiling, Packing density, Contact algorithm, Reduced dimensional model

## Abstract

Endovascular coil embolization is one of the primary treatment techniques for cerebral aneurysms. Although it is a well-established and minimally invasive method, it bears the risk of suboptimal coil placement which can lead to incomplete occlusion of the aneurysm possibly causing recurrence. One of the key features of coils is that they have an imprinted natural shape supporting the fixation within the aneurysm. For the spatial discretization, our mathematical coil model is based on the discrete elastic rod model which results in a dimension-reduced 1D system of differential equations. We include bending and twisting responses to account for the coils natural curvature and allow for the placement of several coils having different material parameters. Collisions between coil segments and the aneurysm wall are handled by an efficient contact algorithm that relies on an octree based collision detection. In time, we use a standard symplectic semi-implicit Euler time stepping method. Our model can be easily incorporated into blood flow simulations of embolized aneurysms. In order to differentiate optimal from suboptimal placements, we employ a suitable in silico Raymond–Roy-type occlusion classification and measure the local packing density in the aneurysm at its neck, wall region and core. We investigate the impact of uncertainties in the coil parameters and embolization procedure. To this end, we vary the position and the angle of insertion of the micro-catheter, and approximate the local packing density distributions by evaluating sample statistics.

## Introduction

Besides from surgical clipping and the use of flow diverters/stents (Briganti et al. 2015; Pierot 2011; Sindeev et al. 2019) and Woven EndoBridge (WEB)-Devices (Goyal et al. 2020; Pierot et al. 2015), endovascular coiling (Pierot and Wakhloo 2013; Guglielmi 2007; Hui et al. 2014; Eddleman et al. 2013) is one of the most commonly used methods (Zhao et al. 2018) in clinical treatment of cerebral aneurysms. It is a volumetric occlusion technique, where the sack of an aneurysm is filled with a thin metal wire, usually platinum, that coils up therein. This causes a stagnation of the blood flow in the aneurysm which together with the intrinsic thrombogenicity of the metal wire leads to embolization (Ngoepe et al. 2018; Byrne et al. 1997).

**Fig. 1:**
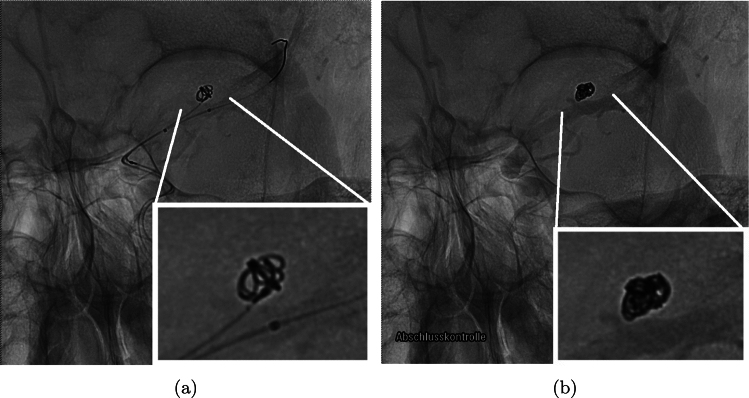
Coiling of an aneurysm at different packing densities. **a** Incomplete occlusion (low packing density), with only framing coil inserted. **b** Completed (high volumetric occlusion) coiling procedure with additional filling coil in place. The images are anonymized and are permitted for distribution. This compliance is ensured by the ethics statement of priority program 2311 of the Deutsche Forschungsgemeinschaft under project number 465242983

The coiling procedure is conducted under continuous imaging supervision in digital subtraction angiography, a fluoroscopy technique, where an interventionist inserts a catheter through a femoral or radial access to reach for the brain along the upstream of blood, carefully moving to the part where the aneurysm is located. Then, via a micro-catheter, the wire is protruded into the aneurysm dome. Coils do exist in various shapes and sizes as well as with various material properties (Ito et al. 2018; Kanenaka et al. 2016; White et al. 2008). A patient-specific choice, e.g., with respect to the length or the imprinted natural shape of the inserted coil(s), is made based on preceding aneurysm measurements as well as the surgeons experience with respect to optimal placement (Neki et al. 2018). Possible choices are, e.g., a stiffer framing coil followed by one or several softer filling and finishing coils depending on the shape of the aneurysm, see Fig. 1. An angiography in combination with the injection of a tracer fluid allows to evaluate the actual occlusion quality and serves as a decision-making tool whether more coiling wires have to be inserted. Typically, a packing density $\psi$, defined as the volume of the inserted coil $V_{{\rm coil }}$ relative to the volume of the aneurysm sack $V_{{\rm aneu}}$, of 20–25% is desired (Sluzewski et al. 2004), with the coil sitting tightly within the aneurysm, not protruding into the parent vessel. As soon as the aneurysm is sufficiently packed, the catheter is retracted from the aneurysm and clot formation is about to begin. The presence of a sufficient amount of coil within the aneurysm reduces the perfusion of the aneurysm sack. This effect is even further enhanced by a growing thrombus, eventually completely shutting of the aneurysm from the bloodstream. On the one hand, a too high packing density may result either in an occlusion of the parent vessel or partial damage to the wall of the aneurysm sack. On the other hand, insufficient packing of the aneurysm bears the risk of either a prolapse within the adjacent vessel or the occurrence of continued blood flow into the aneurysm in a region with poor occlusion. The latter, especially when close to the aneurysm wall, might trigger an aneurysm regrowth (Mascitelli et al. 2015; Kim et al. 2021).

Computational simulations serve as a tool to provide deep insight into the complicated procedures taking place during aneurysm occlusion procedures and subsequent healing mechanisms. In Horn et al. (2018), a system of 28 advection–diffusion–reaction partial differential equations is used to model the clotting induced by bare metal coils in cerebral aneurysms. They show that a more comprehensive model of the clotting cascade results in more realistic thrombus growth when compared to simplified models. The influence of stent-induced vessel deformation on the hemodynamics of intracranial aneurysms is investigated in Sabernaeemi et al. (2023); Salavatidezfouli et al. (2023). Here the computational fluid dynamics methods provide new insight into the flow patterns triggered by stent assisted coiling and show that the deformation of the aneurysm considerably decreases the wall shear stress on the aneurysm wall due to limited entrance of blood into the aneurysm. The occlusion capabilities of memory polyurethane foams in cerebral aneurysms have been simulates in Jarrah et al. (2021). In their study, they develop a model that selects the best fitting memory polyurethane foam for a patient-specific aneurysm by considering thermo-mechanical responses on the foam.

In order to test different coiling configurations, insertion strategies as well as material properties already before the clinical surgery, several mathematical models for coiling (and similar, wirelike) structures have been applied. These models can be grouped roughly into mechanically motivated and phenomenologically motivated ones.

Within the category of phenomenologically motivated models, dynamic path planning algorithms are employed to generate coil distributions in aneurysms. Their major advantage is that they have a relatively low computational complexity. However, they do mostly neglect structure mechanical effects. The method presented in Morales et al. (2012) applies such algorithms to achieve high packing densities of coils, reaching up to ${30\,\mathrm{\%}}$ in aneurysms. In Patel et al. (2021), the authors noted that such methods produce artifacts such as very small loops and kinks that are non-physical. They improved them by developing the *pre-shape path planning algorithm* which considers the natural shape of the coil by including its tendency to minimize the strain energy. This yields more realistic coil placements.

Most of the mechanically motivated models are based on the theory of elastic beams. One of the first models was proposed in Dequidt et al. (2008) and used Timoshenko beam finite elements. This model allows taking curved rest shapes into account and was a first step toward realistic coil simulations. In Babiker et al. (2013), the model is used to generate coil deployments which are then incorporated into CFD simulations. This allowed to analyze the post-treatment hemodynamics and occlusion properties. Since then, several studies have been performed to improve upon the modeling and statistical analysis. The works (Otani et al. 2015, 2020) focus on external forces and internal friction during the coil deployment. Further investigations of post-treatment hemodynamics were made in (Fujimura et al. 2016) to analyze the reduction of the flow velocity triggered by different coil distributions. In Fujimura et al. (2016); Otani et al. (2017); Sheidani et al. (2022); Hariri et al. (2023), the small-scale coil geometry was replaced by a volumetric porosity field in combination with a porous media model reducing the computational cost of a fully resolved free flow simulation. We ultimately aim into a similar direction, i.e., to provide clinicians with a computational tool that allows the realistic simulation of (multiple) coil insertions and their occlusion qualities in silico for enhanced treatment planning, both by geometrical feature analysis of simulated coils in this work, or subsequent hemodynamic simulations including the presence of coils in future works. To reduce the complexity of the mechanical beam element model, a spring–mass system was introduced in Otani et al. (2017). However, it is not possible to include the imprinted natural shape of the coil within this approach.

Our approach is based on the theory of Kirchhoff rods which considers prestresses in the coil that force it into its imprinted shape. To this end, we adapt the “*discrete elastic rods*” (DER) approach from Bergou et al. (2008, 2010) that discretizes the elastic energy functional of a Kirchhoff rod by finite differences. Our model can also be seen as an extension of the simplistic spring–mass model in Otani et al. (2017) and can be easily implemented by the derivative of the discrete energy functional (compare with (Otani et al. 2015; Damiano et al. 2019)). The DER model contains only the minimum degrees of freedom: 3 translational and 1 rotational are needed to represent the natural shape of a coil.

Alternative approaches employ Cosserat rods approximated by Timoschenko beam elements containing 3 translational and 3 rotational degrees of freedom, see Dequidt et al. (2008). Kirchhoff rods assume that axial stretching is negligible, and the Euler–Bernoulli assumption holds. That is, plane sections of a rod remain perpendicular to the neutral axis of the rod while deformation takes place, neglecting longitudinal shear. The Euler–Bernoulli assumption is a good approximation for long slender beams. We neglect axial extension for the following reasons: Real coils have a stretch-resistant filament embedded into their core, preventing them from axial extension. Additionally, the stock wire of a coil is coiled in a manner similar to a helix spring, with a small spacing between loops (approximately $1/10\,D_1$). Axial compression would result in a small deformation in this direction. As soon as the loops of this helical structure touch, the stretch resistance would increase to that of a hollow platinum cylinder and similarly in the extension case to the stretch resistance of the embedded fiber. We assume that at this point, no more axial elongation will take place due to buckling instabilities that result in bending. This assumption is supported by the bounded axial energies due to buckling instabilities in the numerical experiments in Otani et al. (2020).

To extract the natural curvature, the authors of Dequidt et al. (2008) consider a spline curve on the surface of the manufacturing tool given in Wallace et al. (2001), which is an improvement over the approach of using randomized helical loops that are rotated around a ball as done in Babiker et al. (2013). We adapt this approach by modeling the natural curvature of the complex shaped coil as a spline curve that follows approximately the contour given in Wallace et al. (2001).

As in Dequidt et al. (2008), we model the coil’s natural curvature by the twist and curvature of the coil in its natural position. It is important to note that the decomposition of twist and curvature in the natural configuration depends on the chosen local frame, we attach to the coil’s centerline. In the manufacturing process, the natural curvature is first imprinted into the coil wire, and it is afterward retracted into the micro-catheter. Therefore, the question arises on how the coil’s material frames are oriented when it is inside the micro-catheter. Damiano et al. (2019) and Fujimura et al. (2018) take this into account by explicitly retracting the coil, while it is in its natural configuration, into a friction less micro-catheter before simulating the actual coil placement. In our experiments, we have observed that considering the correct local frame when the coil is in the catheter is important, but might be easier to obtain in our case. If relative twist is present when the coil is in the catheter, it will start to unroll once pushed out of the catheter. As we assume that the natural configuration is parametrized by a twist free Bishop frame, the reference twist is zero in this configuration. Therefore, when retracted without friction into a catheter of considerably higher bending stiffness, the minimal energy in (4) is reached when the current twist of local frames is zero as well. This means that the frame in the catheter is also a Bishop frame. With this assumption, the virtual manufacturing in Damiano et al. (2019); Fujimura et al. (2018) can be approximated by imposing a Bishop frame directly in the catheter. We note that this might not hold true if the friction in the catheter is considered and there is an interaction between catheter and coil, as considered in Dequidt et al. 2008.

As in many alternative approaches, we employ an explicit contact algorithm due to the explicit nature of our time integration scheme and the large number of contacts. The frictional coefficients were chosen higher than in other studies (Damiano et al. 2019; Otani et al. 2020) since the vessel walls are rigid in our study. We postulate that an indentation of a vessel wall when a coil loop presses against it provides additional resistance tangential to the wall. For a rigid wall model, we account for this additional resistance by having larger frictional coefficients. Some works employ signed distance function (SDF) for contact detection (Otani et al. 2020). While this method is efficient for detecting coil–wall contacts with the vessel wall, it is not scalable when detecting coil–coil contacts, since the SFD needs to be updated in each time step for the new coil distribution which might be too expensive in terms of computational complexity. In our approach, we use an octree for the efficient search of contacts of the coil. Similar as for the SDF, the octree must be rebuilt in each time step with a complexity of ${\mathcal {O}}(N d)$ where *N* are the number of discrete coil segments and *d* the depth of the octree which is usually much smaller than *N* leading to almost linear complexity.

Our model enables us to simulate a large number of coil deployments in parallel, which will then be used in a subsequent statistical analysis. To perform this analysis, we introduce an in silico Raymond–Roy (Greve et al. 2021; Mascitelli et al. 2015)-type classification for the deployments with respect to their occlusion quality. We account for material and geometric uncertainties by introducing small variations in the coils material parameters as well as in the initial placement location.

The rest of the paper is structured as follows: In Sect. 2, we derive the mathematical model used for our coiling simulations including the data and the numerical methods that are employed. Section 3 then describes our approach toward a statistical occlusion analysis motivated by the Raymond–Roy occlusion classification. Numerical results are presented in Sect. 4 including both specific coil deployment simulations validating our model against actual deployments and results of the statistical analysis. Section 5 contains a discussion of the numerical results, with references to prior research. In Sect. 6, we then give a conclusion of our findings.

## Mathematical modeling

This section starts by introducing the data basis of the three aneurysm shapes that we are using in this study. Following that, we delve into an in depth discussion of the mechanical model used for our coil deployment simulations including its numerical realization and algorithmic aspects.

### Aneurysm geometry

**Fig. 2:**
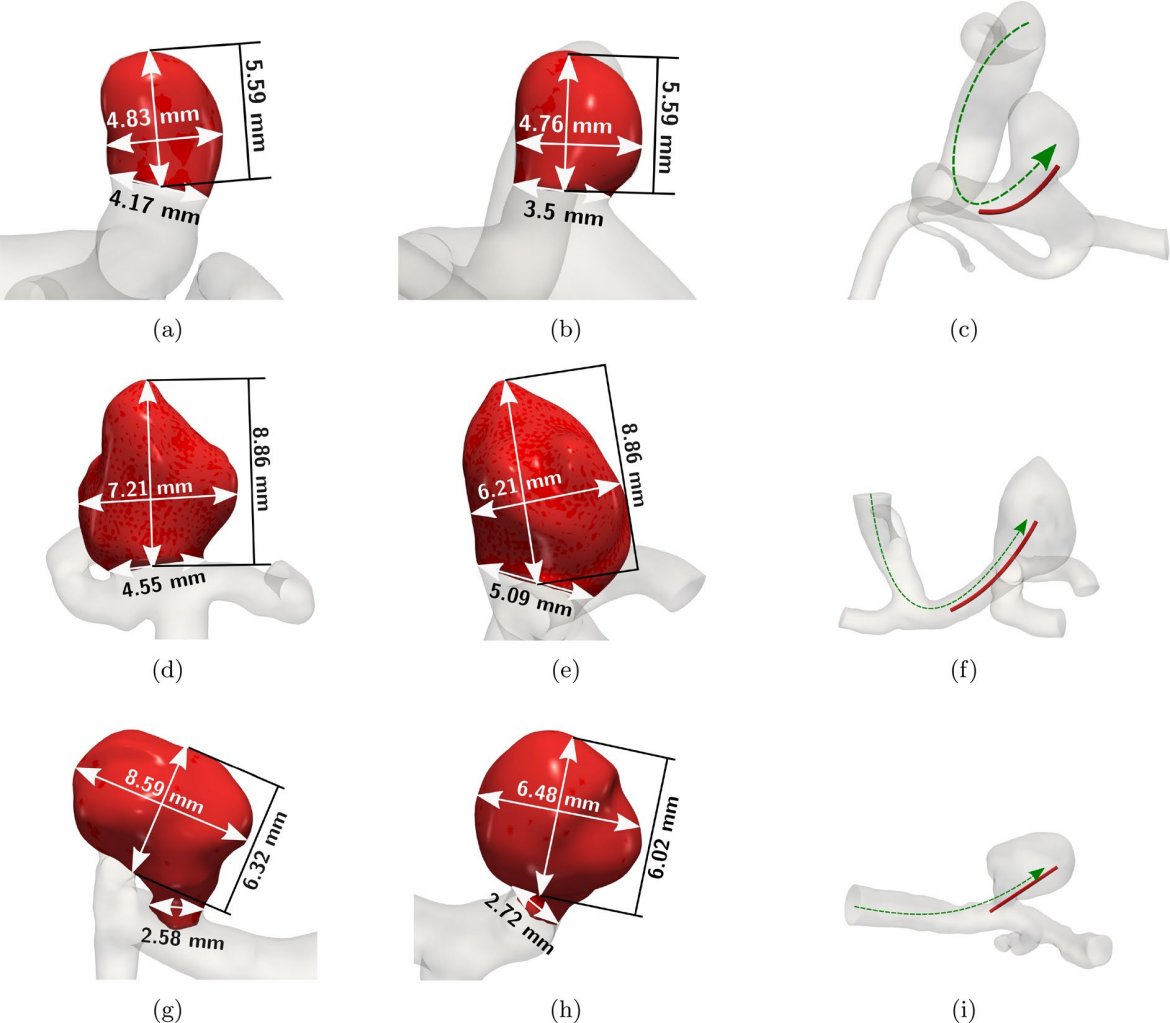
Morphological features of the three aneurysms considered in this study. **a**–**b** are denoted as small aneurysm, **d**–**e** a bifurcation aneurysm and **g**–**e** a narrow neck aneurysm. From the left to the middle column, the perspective is changed by a rotation of 90°. The neck width, dome width and height are given. In the right column, at **c**, **i**, **f**, the insertion direction of the catheter is sketched in green and the position of the catheter is shown in red

In this study, we consider the three aneurysms as given in Fig. 2 which where obtained from Pozo Soler et al. (2017). For convenience of the reader, we have annotated each of the aneurysms by its neck width, dome diameter as well as its height, as it is usually done before an actual clinical intervention. We can classify each of them by their size, see, e.g., Wiebers (2003), with 2–7 mm as very small, 7–12 mm as small and 12–30 mm as large.

The top case in Fig. 2 is an example of a very small aneurysm, the middle case is a bifurcation aneurysm, and an intermediate case is between a very small and a small one. The bottom case stands out since it has a relatively small neck and is in the size range of a small aneurysm. Throughout the paper, we refer to these cases as follows: “*small aneurysm*” for the first one, “*bifurcation aneurysm*” for the second one and “*narrow neck aneurysm*” for the last one mentioned. The geometries where chosen in such a way that they represent sufficiently different but relevant cases to showcase that our coiling model is applicable to a wide range of cases. This means that the dome sizes are chosen with regard to the occurrence frequency when considering treatment studies (Wermer et al. 2007), where the category “*very small*” constitutes the vast majority, followed by “*small*” ones and “*large*” ones being much less frequent.

The raw geometries from the database (Pozo Soler et al. 2017) are associated by default with a triangular surface mesh. For the embolization of the coil, we initially cut off the aneurysm from the parent artery at the approximate position of the neck (see red section in Fig. 2). The coil is then inserted into the separated aneurysm. Finally, after the coil is fully inserted, we enable the full geometry to allow a movement into the direction of the parent artery. To this end, we perform an additional ${10\,\mathrm{\%}}$ of time steps with respect to the total number of time steps, during which we replace the red geometry in Fig. 2 by the full vessel in gray. This assumption is also used in Babiker et al. (2013); Damiano et al. (2019) and accounts for the use of hyperelastic balloons in challenging coiling scenarios, specially large-necked aneurysms. For the modification of the mesh, we use blender^1^ and for the re-meshing meshlab.^2^ The micro-catheter, through which the coil is pushed into the aneurysm, is considered as an obstacle in our simulation. We model it as a B-spline consisting of three nodes. The position of the micro-catheter is fixed as shown in Fig. 2 (right column) if not stated otherwise.

### Strain energy in elastic rods

**Fig. 3:**
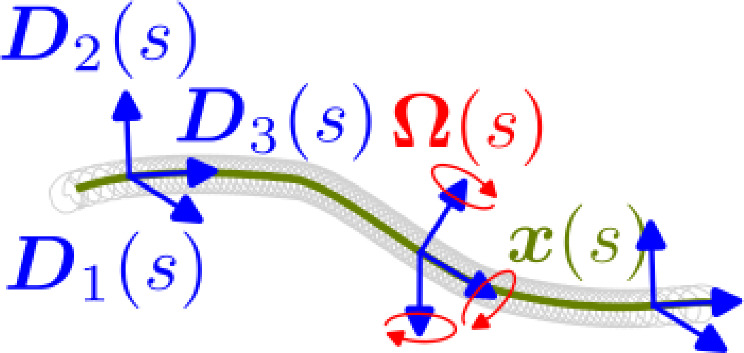
A space curve ${\varvec{x}}$ parametrized by its arc length *s* together with the material directors ${\varvec{D}}_{1}(s), {\varvec{D}}_{2}(s), {\varvec{D}}_{3}(s),$ and their corresponding Darboux vector $\varvec{\Omega }(s)$

We assume that the coil can be modeled by Kirchhoff’s theory of elastic rods as presented in Audoly and Pomeau (2000). Herein a three-dimensional space curve ${\varvec{x}}:[0,L]\rightarrow {\mathbb {R}}^3,~s\mapsto {\varvec{x}}(s)$ with arc-length parameter $s\in [0,L]$ is used to trace the centerline of a rod (see Fig. 3). For each position on the space curve, we attach a right-handed frame of orthonormal directors ${\varvec{D}}_{1}(s), {\varvec{D}}_{2}(s)\text{ and }{\varvec{D}}_{3}(s)$, each $\in {\mathbb {R}}^3$ used as columns to form the matrix $\big \{{\varvec{D}}_{1}(s), {\varvec{D}}_{2}(s), {\varvec{D}}_{3}(s)\big \}\in {\text {SO}}(3)$. They represent the orientation of the rods cross section and are material to the curve; hence, they are called material directors. As shown in Eddleman et al. (2013), an extension of the coil while surgery may lead to serious complications and has to be suppressed by the design of the coil. (Here non-elastic filaments are embedded in its core and fixed at both ends of the coil, see Fig. 6b.) Thus, we assume that our rod is inextensible, with $\Vert {\varvec{x}}'(s)\Vert =1$. Further the material frame is assumed to be adapted, i.e., ${\varvec{D}}_{3}(s) = {\varvec{x}}'(s)$ at each position *s*. (The arc-length derivative is denoted by $\partial (\cdot )/\partial s=(\cdot )'.$) This expresses that no shear occurs and implies that bending and twisting are the most significant modes of deformation. A standard concept from differential geometry is the Darboux vector $\varvec{\Omega} (\varvec{s})$ defined by

1 $$\begin{aligned} {\varvec{D}}_i'(s) = \varvec{\Omega }(s) \times {\varvec{D}}_i(s) \quad \forall i \in \{1,2,3\} \end{aligned}$$

which can be represented as $\varvec{\Omega }(s) = \kappa _1(s) {\varvec{D}}_1(s) + \kappa _2(s) {\varvec{D}}_2(s) + \tau (s) {\varvec{D}}_3(s)$; we refer to (Audoly and Pomeau 2000). We interpret $\varvec{\Omega }(s)$ as the rotation velocity around the directors. By inserting $\varvec{\Omega }(s)$ into the Frenet–Serret equations (Audoly and Pomeau 2000), the elastic strains for the curvature $\varvec{\kappa }=(\kappa _1,\kappa _2)^T$ and the torsion/twist $\tau$ can be obtained from (1) as

2 $$\begin{aligned} {\varvec{D}}'_3 \cdot {\varvec{D}}_1 = - \kappa _2, {\varvec{D}}'_3 \cdot {\varvec{D}}_2 = \kappa _1, {\varvec{D}}'_2 \cdot {\varvec{D}}_1 = \tau . \end{aligned}$$

Here we applied the identity ${\varvec{a}}\cdot ({\varvec{b}}\times {\varvec{c}}) = {\varvec{b}}\cdot ({\varvec{c}}\times {\varvec{a}})$. Having expressions for the elastic strains, we are in the position to formulate the elastic potential of the rod as

3 $$\begin{aligned} E({\varvec{x}}) = \frac{1}{2}\int \limits _{0}^L \varvec{\kappa }^T{\varvec{B}}\varvec{\kappa } + \beta \tau ^2 {{\text{d}}\textit{s}}, \end{aligned}$$

where ${\varvec{B}}\in {\mathbb {R}}^{2\times 2}$ and $\beta \in {\mathbb {R}}$ are dependent on the material parameters and the geometry of the cross section.

**Fig. 4:**
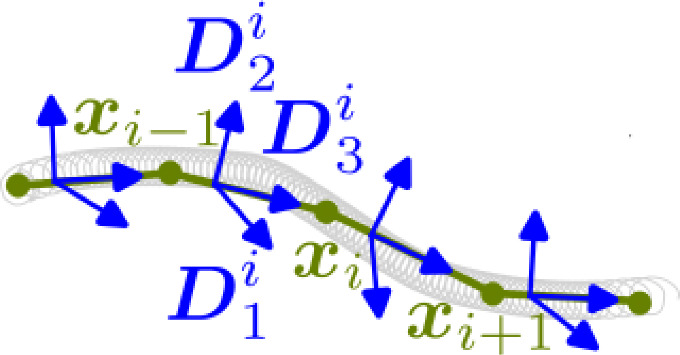
A discrete space curve with material points ${\varvec{x}}_i$ for $i \in \{0,1,...,N-1\}$ and edges ${\varvec{e}}^j={\varvec{x}}_{j+1}-{\varvec{x}}_{j}$ for $j \in \{0,...,N-2\}$ on which the material frames ${\varvec{D}}_{1}^j, {\varvec{D}}_{2}^j, {\varvec{D}}_{3}^j$ are attached

From this point on, it is standard to obtain the continuous Kirchhoff equations for the equilibrium of an elastic rod by a variational argument, see, e.g., Audoly and Pomeau (2000). We follow the approach of Bergou et al. (2008, 2010) called “discrete elastic rods" (DER) which discretizes the strain energy directly. Many details are omitted in our short review of the DER, but refer the interested reader to a recent primer on the topic (Jawed et al. 2018). We start by discretizing the rod as a finite set of *N* material points ${\varvec{x}}_i:= {\varvec{x}}(s_i)$ for $i\in \{0,1,...,N-1\}$, where two subsequent points are connected by an edge ${\varvec{e}}^i={\varvec{x}}_{i+1}-{\varvec{x}}_i$ (see Fig. 4), following the notation of Bergou et al. (2008, 2010), and we denote node quantities by subscript indices and edge quantities by superscript indices. For each edge, we define one material frame $\{ {\varvec{D}}_1^i, {\varvec{D}}_2^i, {\varvec{D}}_3^i \} \in {\text {SO}}(3)$ for $i\in \{0,1,...,N-2\}$, where the constraint ${\varvec{D}}_3(s)=x'(s)$ is explicitly fulfilled by letting the third director be the tangent ${\varvec{t}}^i:={\varvec{e}}^i/\Vert {\varvec{e}}^i \Vert = {\varvec{D}}_3^i$ for all $i\in \{0,1,...,N-2\}$. The inextensibility constrained can be satisfied by adding an axial strain component in the stretching energy, see (4). The strain energy in (3) considers only rods that are pre-shaped straight when no external forces act. For medical coils, it is important to drop this assumption since they are naturally curved (Eddleman et al. 2013; Wallace et al. 2001; Ito et al. 2018; White et al. 2008). For our upcoming discussion, barred quantities are used to refer to the curve in its natural position $\overline{{\varvec{x}}}$ while quantities that are not barred refer to the curve in deformed configurations. We denote by $\overline{{\varvec{e}}}^j=\overline{{\varvec{x}}}_{j+1}-\overline{{\varvec{x}}}_{j}$ the edge vector in the natural position and by ${\overline{l}}_i=1/2(\Vert \overline{{\varvec{e}}}^{i-1}\Vert + \Vert \overline{{\varvec{e}}}^{i}\Vert )$ the Voronoi length created by two consecutive edges in the natural position. A curve ${\varvec{x}}$, which is not straight in its natural curved position $\overline{{\varvec{x}}}$, can have a curvature $\overline{\varvec{\kappa }}$ and twist ${\overline{\tau }}$ different from the current curvature and twist $\varvec{\kappa }, \tau$. This allows to write the enriched discrete strain energy in the following way

4 $$\begin{aligned} E_{\textrm{tot}}&= \sum \limits _{j=0}^{n-2} \frac{1}{2}\alpha \big (\frac{\Vert {\varvec{e}}^j\Vert }{\Vert \overline{{\varvec{e}}}^j\Vert } - 1\big )^2 \Vert \overline{{\varvec{e}}}^j\Vert \nonumber \\&+\quad \sum \limits _{i=1}^{n-2} \frac{1}{2 {\overline{l}}_i}\left( \varvec{\kappa }_i - \overline{\varvec{\kappa }}_i\right) {\varvec{B}} \left( \varvec{\kappa }_i - \overline{\varvec{\kappa }}_i\right) ^T \nonumber \\&+ \quad \sum \limits _{j=1}^{n-2} \frac{1}{2 {\overline{l}}_j} \beta \left( \tau ^j-{\overline{\tau }}^j\right) ^2, \end{aligned}$$

where the first term corresponds to the axial strain energy ensuring with $\alpha >0$ chosen sufficiently large the inextensibility of the rod (see Appendix C). To find expressions for $\varvec{\kappa }_i$ and $\tau ^i$, we note that since ${\varvec{D}}_3^i$ is adapted to the curve, the directors ${\varvec{D}}_1^i, {\varvec{D}}_2^i$ form a plane to which the curvature binormal $(\kappa {\varvec{b}})$ is coplanar. By straight forward calculations as, demonstrated in (Jawed et al. 2018), one can find an expression for $(\kappa {\varvec{b}})$ at the node *i* by:

5 $$\begin{aligned} (\kappa {\varvec{b}})_i = \frac{2 {\varvec{t}}^{i-1}\times {\varvec{t}}^{i}}{1+{\varvec{t}}^{i-1}\cdot {\varvec{t}}^{i}}. \end{aligned}$$

This expression is referred to as nodal discrete integrated curvature binormal. With help of $(\kappa {\varvec{b}})_i$, one derives the discrete nodal curvatures

6 $$\begin{aligned}&\kappa _{i1} = \frac{1}{2} \big ({\varvec{D}}_2^{i-1} + {\varvec{D}}_2^{i}\big ) \cdot (\kappa {\varvec{b}})_i,\nonumber \\&\kappa _{i2} = -\frac{1}{2} \big ({\varvec{D}}_1^{i-1} + {\varvec{D}}_1^{i}\big ) \cdot (\kappa {\varvec{b}})_i. \end{aligned}$$

We note that this is the finite difference formulation for the curvatures in (2) since

7 $$\begin{aligned} \lim \limits _{{\overline{\ell }}\rightarrow 0} \frac{ {\varvec{t}}(s-{\overline{\ell }}/2)\times {\varvec{t}}(s+{\overline{\ell }}/2))}{{\overline{\ell }}} ={\varvec{t}}'={\varvec{D}}'_3. \end{aligned}$$

**Fig. 5:**
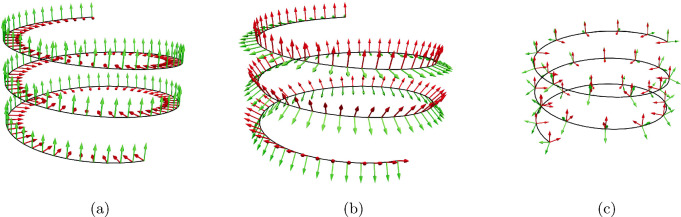
Framing of a helix where ${\varvec{D}}_1$ is represented in green and ${\varvec{D}}_2$ in red (**a**) An example for a material Frame with the director ${\varvec{D}}_1$ in green and ${\varvec{D}}_2$ in red (**b**) Bishop Frame with directors ${\varvec{U}}_1$ in green and ${\varvec{V}}_2$ in red. **c** In red, we show a material Frame of directors ${\varvec{D}}_1,{\varvec{D}}_2$ and in green a Bishop frame for the curve ${\varvec{U}}_1,{\varvec{V}}_2$. Since the Bishop frame is twist free, we can rotate it on each edge into the material frame to get the absolute reference angle with respect to the Bishop frame. On two consecutive edges $(j,j+1)$, the difference in this angle is the twist of the material frame $\tau ^j$

For the discrete torsion, we first introduce the parallel transport given by the rotation matrix ${\varvec{R}}_{{\varvec{t}}^{j-1}}^{{\varvec{t}}^{j}}$ that rotates the tangent of edge $j-1$ into the tangent of edge *j*. An explicit representation of the matrix follows from the Rodrigues formula (Dai 2015):

8 $$\begin{aligned}&{\varvec{R}}_{{\varvec{t}}^{j-1}}^{{\varvec{t}}^{j}} = {\varvec{I}} + {\varvec{K}} + {\varvec{K}}^2\frac{1}{1-({\varvec{t}}^{j-1}\cdot {{\varvec{t}}^{j})}} \nonumber \\&\text { with } {\varvec{K}}{\varvec{v}}=({\varvec{t}}^{j-1}\times {{\varvec{t}}^{j})}\times {\varvec{v}} \text { for } {\varvec{v}}\in {\mathbb {R}}^3. \end{aligned}$$

In Bergou et al. (2008), the twist degree of freedom is parametrized with respect to the Bishop frame (Bishop 1975) $\{{\varvec{U}}^j,{\varvec{V}}^j,{\varvec{D}}_3^j\}$. It is for any continuous space curve uniquely defined provided ${\varvec{U}}^0,{\varvec{V}}^0$ is fixed, see Fig. 5 which shows the frames on a helix. Applying the parallel transport operator, we find

9 $$\begin{aligned} {\varvec{U}}^{j} = {\varvec{R}}_{{\varvec{t}}^{j-1}}^{{\varvec{t}}^{j}}{\varvec{U}}^{j-1},\quad {\varvec{V}}^{j} = {\varvec{R}}_{{\varvec{t}}^{j-1}}^{{\varvec{t}}^{j}}{\varvec{V}}^{j-1}. \end{aligned}$$

In Fig. 5, the difference between a frame that has a nonzero twist and a torsion free Bishop frame is shown. In case of the frame that has nonzero twist, the first director points into the helix center for any point on the helix, which is only possible when then frame is rotated by a constant angle increment while traversing it. For the Bishop frame, we choose the first director at the lower end of the helix and then propagate it by (8). This does not introduce any twist but causes the Bishop frame to turn upwards while propagating it along the curve. One can show that the Bishop frame has always zero twist and can be used as a reference frame to measure the twist in the material directors $\{{\varvec{D}}_1^j,{\varvec{D}}_2^j,{\varvec{D}}_3^j\}$. Therefore, we first measure the signed angle between either ${\varvec{D}}_1^j$ and ${\varvec{U}}^j$ or ${\varvec{D}}_2^j$ and ${\varvec{V}}^j$ which we call $\phi ^j$. In a second step, we calculate the discrete integrated twist as the increment $\tau _j=\phi ^j-\phi ^{j-1}$. This increment can also be directly expressed as the angle increment to align ${\varvec{D}}_i^{j}$ with ${\varvec{R}}_{{\varvec{t}}^{j-1}}^{{\varvec{t}}^{j}}{\varvec{D}}_i^{j-1}$ around ${\varvec{D}}_3^{j}$ for $i\in \{1,2\}$ such that we have

10 $$\begin{aligned} {\varvec{D}}_1^{j} = \cos (\tau ^j){\varvec{R}}_{{\varvec{t}}^{j-1}}^{{\varvec{t}}^{j}}{\varvec{D}}_1^{j-1} +\sin (\tau ^j){\varvec{R}}_{{\varvec{t}}^{j-1}}^{{\varvec{t}}^{j}}{\varvec{D}}_2^{j-1},\nonumber \\ {\varvec{D}}_2^{j} = \cos (\tau ^j){\varvec{R}}_{{\varvec{t}}^{j-1}}^{{\varvec{t}}^{j}}{\varvec{D}}_2^{j-1} -\sin (\tau ^j){\varvec{R}}_{{\varvec{t}}^{j-1}}^{{\varvec{t}}^{j}}{\varvec{D}}_1^{j-1}. \end{aligned}$$

To close the discretization of the elastic energy, it remains to formulate expressions for the natural curvature and twist $\overline{\varvec{\kappa }_i}$ and ${\overline{\tau }}_i$ of the rod, respectively. To this end, we employ again the Bishop frame as the material directors of the discrete curve ${\varvec{x}}$ in its natural shape $\overline{{\varvec{x}}}_i$. In that way, $\{\overline{{\varvec{D}}}_1^{j},\overline{{\varvec{D}}}_2^{j},\overline{{\varvec{D}}}_3^{j}\}$ coincide with the Bishop frame of $\overline{{\varvec{x}}}_i$. This allows us to measure the natural curvatures by (6), and moreover, we note that the twist for this configuration is zero. We point out that the Bishop frame is not the only possible framing for the curve in its natural position. In fact, any frame will lead to the same equilibrium position of the rod when minimizing the energy potential in (4) as shown in Bertails-Descoubes et al. (2018).

### The coil dynamics

To model the dynamics of the rod, we apply Newtons second law which yields a system of ordinary differential equations

11 $$\begin{aligned} \begin{bmatrix} {\varvec{M}}\ddot{{\varvec{X}}} \\ {\varvec{0}} \end{bmatrix} + \begin{bmatrix} {\varvec{D}}_{{\varvec{X}}} \dot{{\varvec{X}}}\\ {\varvec{D}}_{\varvec{\Phi }} \dot{\varvec{\Phi }} \end{bmatrix} = \underbrace{ -\begin{bmatrix} \nabla _{{\varvec{X}}} E_{\textrm{tot}} \\ \nabla _{\varvec{\Phi }} E_{\textrm{tot}} \end{bmatrix} }_{{\varvec{F}}_{\textrm{int}}} + \begin{bmatrix} {\varvec{F}}_{\textrm{ext}} \\ {\varvec{0}} \end{bmatrix}, \end{aligned}$$

with the vector $$ {\varvec{X}}=\big ({\varvec{x}}_0,{\varvec{x}}_1,...,{\varvec{x}}_{N-2},{\varvec{x}}_{N-1} \big )$$ containing the translational degrees of freedom (DOFs) and $$\varvec{\Phi }=\big (\varvec{\phi }_0,\varvec{\phi }_1,...,\varvec{\phi }_{N-2} \big )$$ containing the rotational DOFs. $${\varvec{F}}_{\textrm{ext}}$$ are the external forces and moments due to wire–wire and wire–aneurysm–surface collisions. Expressions for $$\nabla _{{\varvec{X}}} E_{\textrm{tot}}$$ are derived in Jawed et al. (2018), and $$\nabla _{\varvec{\Phi }} E_{\textrm{tot}}$$ can be found in Appendix A. $${\varvec{M}}$$ and $${\varvec{D}}_{{\varvec{X}}}, {\varvec{D}}_{\varvec{\Phi }} $$ are the mass and damping matrices for translational and rotational DOFs, respectively. We assume that they are diagonal and set $${\varvec{M}}=m{\varvec{I}}$$, $${\varvec{D}}_{{\varvec{X}}}=\eta _{{\varvec{X}}}{\varvec{I}}$$, $${\varvec{D}}_{\varvec{\Phi }}=\eta _{\varvec{\Phi }}{\varvec{I}}$$ with $${\varvec{I}}$$ denoting the identity matrix and *m*, $$\eta _{{\varvec{X}}},\eta _{\varvec{\Phi }}$$ denoting the masses that are lumped into the vertices and scalar damping coefficients. Note that we have set the mass matrices for the rotational DOFs to zero. To motivate this simplification, we assume that the node mass of the elastic rod is *m* then the rotational moment of inertia is proportional to $$m D_2^2 $$ making it excessively small due to the small diameter $$D_2$$; hence, we neglect it. In Bergou et al. (2008), a similar simplification is applied with the difference that there the rotational moment and the rotational damping are set to zero resulting in $$\nabla _{\varvec{\Phi }} E_{\textrm{tot}}=0$$. To ensure this constraint, one needs to optimize in each time step of the numerical method the energy with respect to the rotational DOFs which is costly. In our implementation, we add the diagonal damping matrix $${\varvec{D}}_{\varvec{\Phi }}$$ which when chosen sufficiently small approximates the condition $$\nabla _{\varvec{\Phi }} E_{\textrm{tot}}=0$$. Further this enables us to circumvent the minimization of $$E_{\textrm{tot}}=0$$ with respect to $$\varvec{\Phi }$$. To solve the ODE system, we apply the symplectic Euler method (Hairer et al. 2006) as time stepping scheme

12a $$\begin{aligned} {\varvec{M}}\dot{{\varvec{X}}}(t_{n+1})&= \Delta t \big ( - \nabla _{{\varvec{X}}} E_{\textrm{tot}}(t_{\textrm{n}})+ \nonumber \\&{\varvec{F}}_{\textrm{ext}}-{\varvec{D}}_{{\varvec{X}}}(t) \dot{{\varvec{X}}}(t_{\textrm{n}}) \big ) + {\varvec{M}}\dot{{\varvec{X}}}(t_{\textrm{n}}), \end{aligned}$$

12b $$\begin{aligned} \begin{bmatrix} {\varvec{X}}(t_{n+1})\\ \varvec{\Phi }(t_{n+1}) \end{bmatrix}&= \Delta t \begin{bmatrix} \dot{{\varvec{X}}}(t_{n+1}) \\ - {\varvec{D}}_{\varvec{\Phi }}^{-1}\nabla _{\varvec{\Phi }} E_{\textrm{tot}}(t_{\textrm{n}}) \end{bmatrix} + \begin{bmatrix} {\varvec{X}}(t_{\textrm{n}})\\ \varvec{\Phi }(t_{\textrm{n}}) \end{bmatrix}. \end{aligned}$$

where $\Delta t:=t_{n+1}-t_{\textrm{n}}$ refers to the time step size of the discrete time steps $t_{\textrm{n}}$ with $n \in \{0,..., {N_T}-1\}$ and $N_T\Delta t=T$. Note that for the symplectic Euler method, we first solve (12a) yielding $\dot{{\varvec{X}}}(t_{n+1})$ that is then used in (12b).

### Boundary/initial conditions for endovascular coiling

Here we formulate appropriate boundary conditions that approximate the real endovascular coiling procedure. We assume that the coil is pushed out of the micro-catheter at a constant speed ${\varvec{v}}_{\textrm{push}}$. Let $I_{\textrm{micro}}$ be the set of node indices such that the corresponding nodes are still inside the micro-catheter at time $t_{\textrm{n}}$. Then for any node with index $i\in I_{\textrm{micro}}$, we set the velocity as $\dot{{\varvec{x}}}_i={\varvec{v}}_{\textrm{push}}$ and for any node with index $i\not \in I_{\textrm{micro}}$, the velocity can be calculated from (12a)–(12b). In the rotational degrees of freedom, we assume that the coil can freely rotate due to the lubrication therein. Note that this cannot lead to a blowup in the angular velocity since we have applied the damping ${\varvec{D}}_{\Phi }$. We assume do nothing boundary conditions for the free ends of the coil. To do so, we apply different forces and moments on the first and last node and edge, respectively. The forces are stated in Jawed et al. (2018) and the moments can be found in Appendix A. As initial condition, we assume that the coil is placed in a straight configuration in front of the micro-catheter inlet pointing into the direction of the insertion velocity ${\varvec{v}}_{\textrm{push}}/\Vert {\varvec{v}}_{\textrm{push}}\Vert$. Additionally, the natural curve for the centerline of the coil is parametrized by a Bishop frame. Further we assume that the initial orientation of the directors is given as a twist free Bishop frame.

### Elastic spring constants of coils

**Fig. 6:**
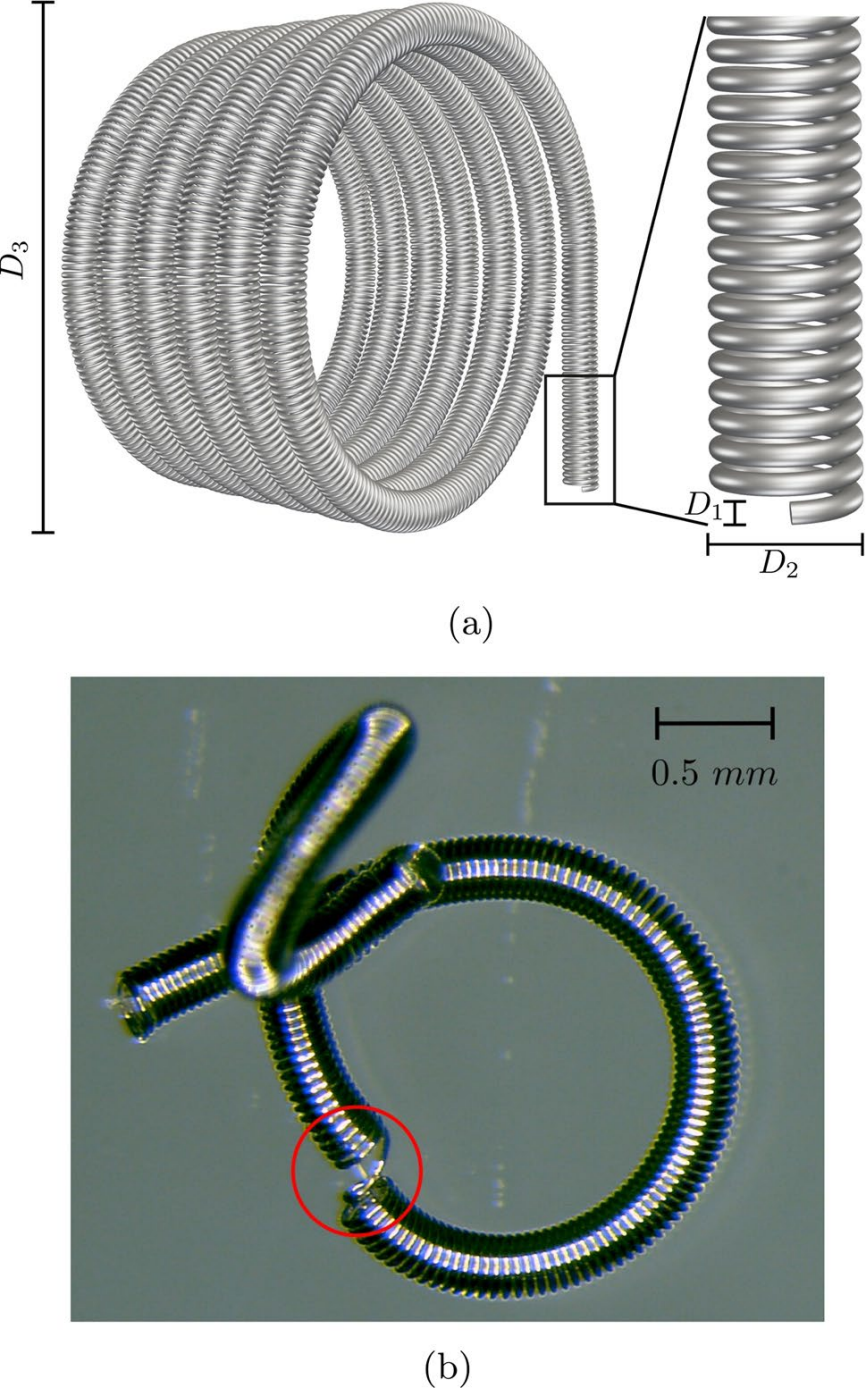
**a** Characteristic lengths $D_1, D_2$ and $D_3$ of the coil with a natural shape corresponding to a helix. **b** Photograph of a damaged coil segment. At the red circled section, the helical structure was forcefully opened to show the filament at the centerline

The material parameters ${\varvec{B}}=b{\varvec{I}}$ and $\beta$ in the discrete energy potentials (4) can be obtained by integration over the rod cross section as described in Jawed et al. (2018). Considering the coil structure in detail, one identifies three characteristic length scales as shown in Fig. 6a. The first one called $D_1$ is the diameter of the coil stock wire which is then coiled up into a helical shape with radius $D_2$. The helical shape essentially makes up the coil. The natural curvature is then imprinted into the $D_3$ diameter. In Fig. 6b, a photograph of a coil segment is depicted. In our coiling model, we do not resolve the details of the stock wire. We rather consider the helical structure with $D_2$ as a rod and express the shape of the stock wire via the constants $b,\beta$. This can be done by calculating the bending and twisting stiffness for a torsion string as done in Wahl and Bisshopp (1964, Chapter 9,17), Otani et al. (2020) which yields the following expressions

13 $$\begin{aligned}{} & {} b = \frac{E_{{w}} D_1^4p_c}{32\,(2+\mu _{{w}}) D_2},{} & {} \beta = \frac{E_{{w}} D_1^4p_c}{64\,D_2}.{} & {} \end{aligned}$$

In these, $E_{{w}}$ and $\mu _{{w}}$ denote the stock wires Young’s modulus and Poisson ratio. The quantity $p_c$ is the distance between the centerline of each turn in the $D_2$ structure and defined by $p_c=D_1 p$ where $p>1$ is often refereed to as the pitch (White et al. 2008). In the remainder of this work, we set $p=1.1$. Note that the formulas (13) are derived with the assumption that the coil’s helix structure in $D_2$ is not excessively deformed, and the loops do not touch. This might not be the case in all simulations, but we assume that formulas (13) approximate the real stiffnesses better than other stiffer geometries such as a hollow cylinder.

### Contact algorithm: collision detection

The contacts in our simulation can be split up into contacts of the coil with the aneurysm wall and contacts of the coil with itself. This section describes how contacts are identified and collisions are modeled.

Naively one can check for a coil with *N* edges and a wall triangulated by *M* facets if a coil self-collision occurs in ${\mathcal {O}}(N^2)$ and if a coil–wall collision occurs in ${\mathcal {O}}(NM)$. Since *N* and *M* are usually relatively large (at least $10^3$), a naive approach increases the computational complexity. Therefore, more advanced methods have been proposed (Eberly 2006; Jiménez et al. 2001) that only consider contacts in close vicinity by dividing the simulation domain into subsections. In this study, we use the octree method (Behley et al. 2015) that belongs to this class of methods. The simulation domain $\Omega$ is partitioned into a finite set of intervals; each of it roughly containing the same number of contact objects. Crucial about the method is that the hierarchy of intervals can be represented in a tree structure. Therefore, the search for a contact partner of a certain object can be restricted to the partners in a specific interval which is found in the tree structure in logarithmic time complexity. In our case, the time complexity for coil–wall collision detection is reduced to ${\mathcal {O}}(N\log (M))$ and, respectively, for coil self-collision detection to ${\mathcal {O}}(N\log (N))$.

**Fig. 7:**
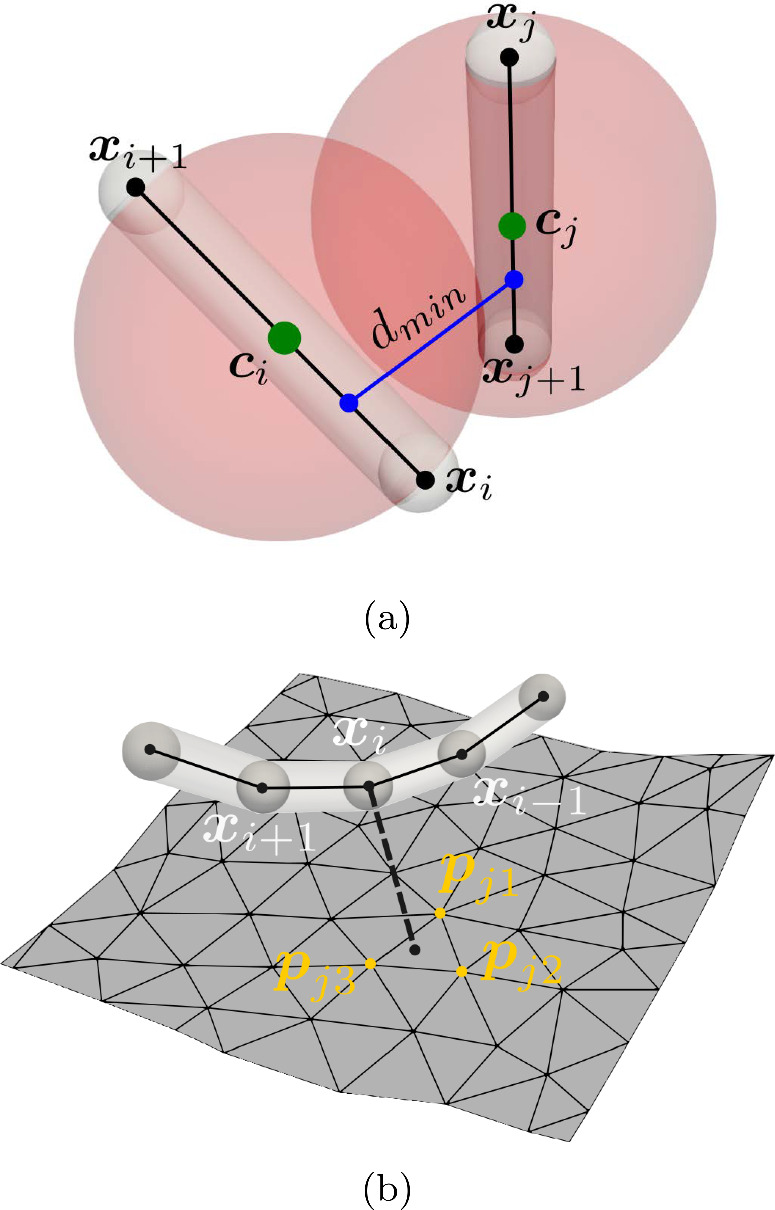
**a** Setup for collision detection between edge segments. **b** Setup for collision detection between an edge and an triangular surface element. By $d_{\textrm{min}}$ we denote the minimal scalar distance between centerlines of the contact partners

Our collision detection is structured in two phases.

1. Evaluate a simple necessary condition that allows to find potential collision partners by querying the octree.
2. For all potential collision partners, evaluate the minimum distance between them to discover all collisions and contact points.

We start by formulating a necessary condition for coil self-collisions, see Fig. 7a. For each edge ${\varvec{e}}^i$ on the coil, we create a sphere with center ${\varvec{c}}^i = ({\varvec{x}}_i+{\varvec{x}}_{i+1})/2$ and some radius. The radius is then chosen in such a way that whenever another edge center ${\varvec{c}}_j$ is located in the sphere of ${\varvec{c}}_i$, we have a necessary condition for a collision between the two edges. We set the sphere’s radius to the diagonal distance of the triangle with side lengths ${\overline{\ell }}/2$ and $D_2/2$. Therefore, we can express the condition as

14 $$\begin{aligned} \Vert {\varvec{c}}_j-{\varvec{c}}_i\Vert \le \sqrt{({\overline{\ell }}/2)^2 + D_2^2}. \end{aligned}$$

Next we verify whether a collision between edge ${\varvec{e}}^i$ and the potential partner ${\varvec{e}}^j$ is actually occurring by calculating the minimum distance vectors ${\varvec{d}}^{ij}_{\textrm{min}}$ between the segments $s_i = \{ \alpha {\varvec{e}}^i +{\varvec{x}}_{i}:\alpha \in [0,1]\}$ and $s_j = \{ \alpha {\varvec{e}}^j +{\varvec{x}}_{j}:\alpha \in [0,1]\}$ by the method proposed in Lumelsky (1985). Finally, a collision takes actually place if

15 $$\begin{aligned} d_{\textrm{min}}^{ij} = \Vert {\varvec{d}}^{ij}_{\textrm{min}} \Vert \le D_2. \end{aligned}$$

Next the conditions for wall collisions are illustrated in Fig. 7b. We assume that the wall surface is meshed by triangles $T_j\in {\mathcal {T}}$, which are defined by their vertices ${\varvec{p}}_{j1}, {\varvec{p}}_{j2}, {\varvec{p}}_{j3}$. To formulate a necessary condition for a wall collision, we again consider the center point of an edge ${\varvec{c}}_i$ and the center point of a triangle ${\varvec{c}}_{T_j}=({\varvec{p}}_{j1}+{\varvec{p}}_{j2}+ {\varvec{p}}_{j3})/3$. Let $r_{ST}$ be the radius and center ${\varvec{c}}_{T_j}$ of the smallest sphere that contains all $T_i\in {\mathcal {T}},$ then a sufficient condition for a collision is

16 $$\begin{aligned} \Vert {\varvec{c}}_i - {\varvec{c}}_{T_j} \Vert \le D_2/2 + r_{ST}. \end{aligned}$$

To check between each partner whether a collision occurs or not, we first project the point ${\varvec{c}}_i$ onto the plane defined by the triangle $T_j$ with surface normal ${\varvec{n}} = ({\varvec{p}}_{i1}- {\varvec{p}}_{i2}) \times ({\varvec{p}}_{i1}- {\varvec{p}}_{i3})$ and denote this projection by ${\varvec{c}}_{i,T_j}$. For the projected point, we check whether it is contained in the triangle ${\varvec{c}}_{i,T_j}\in {T_j}$. If it is contained in the triangle, we calculate the minimum distance by $\Vert {\varvec{c}}_{i,T_j}-{\varvec{c}}_{i}\Vert$. In all other cases, no collision takes place. Note that our model assumes that no contact partner protrudes into the wall.

### Contact algorithm: friction model

Our collision model is based on the one used in Gazzola et al. (2018) which is a Coulomb stick slip friction contact model. Although being local, the Coulomb friction model is widely used. Upon contact, a virtual contact plane is formed between the contact partners. The repulsive force in normal direction resulting from the non-penetration condition constitutes an upper bound for the tangential force. As long as the magnitude of it is below a threshold, the two contact partners stick together in tangential direction and there is no sliding. Once the tangential force reaches the upper limit, sliding starts to counterbalance the tangential force. The mathematical model, in contrast to a simplified Tresca friction model, results in a quasi variational inequality, i.e., the feasible set depends on the solution itself. We start by describing the forces that act in normal direction. Assuming a collision takes place between the edges $(i,i+1)$ and $(j,j+1)$, we find the minimal distance between their cylindrical hulls as $d^{ij}_{\textrm{min}}$. Since a collision takes place, we can calculate the overlap by $\epsilon _{ij}=D_2 - d^{ij}_{\textrm{min}}$. Then a collision of the coil segment on edge $(i,i+1)$ with the coil segment of edge $(j,j+1)$ introduces the following force in collision direction

17 $$\begin{aligned} ({\varvec{F}}_{\hbox {CC},\perp }^{i})_j&= -H(\epsilon _{ij})\cdot \big ( k_{\textrm{sc}}\epsilon _{ij} \nonumber \\&+\gamma _{\textrm{sc}}(\dot{{\varvec{x}}}_{i,i+1} - \dot{{\varvec{x}}}_{j,j+1})\cdot {\varvec{d}}^{ij}_{\textrm{min}}\big ){\varvec{d}}^{ij}_{\textrm{min}}. \end{aligned}$$

Here $\dot{{\varvec{x}}}_{i,i+1}$ is the linear interpolation of the nodal velocities onto the point of collision on the edge $(i,i+1)$ and $\dot{{\varvec{x}}}_{j,j+1}$, respectively. The model is activated upon collision by the Heaviside function $H(\epsilon _{ij})$. The coefficient $k_{\textrm{sc}}$ stands for the coil self-contact spring constant and $\gamma _{\textrm{sc}}$ for the coefficient of dissipation. Recall that the vector ${\varvec{d}}_{\textrm{min}}^{ij}$ is the minimum distance vector.

Next we formulate the wall collisions by decomposing the nodal forces and velocities into a wall tangential and wall orthogonal part $(-\nabla _{{\varvec{X}}}E_{\textrm{tot}})_i+\sum _{j=1}^{n}({\varvec{F}}_{\hbox {CC}}^i)_j={\varvec{F}}_{\perp } \oplus {\varvec{F}}_{\Vert }$ and ${\varvec{v}}={\varvec{v}}_{\perp } \oplus {\varvec{v}}_{\Vert }$. Assuming a collision takes place between a triangle $T_k\in {\mathcal {T}}$ and a node *i* with intersection width $\epsilon = D_2-d_{\textrm{min}}^{ij}$, then the normal force due to the collision is

18 $$\begin{aligned} ({\varvec{F}}^i_{\hbox {CW},\perp })_k = -H(\epsilon )\cdot \big (&\Vert {\varvec{F}}_{\perp }\Vert +k_{w}\epsilon _{ij} \nonumber \\&+\gamma _{w}\dot{{\varvec{x}}}_{i}\cdot {\varvec{n}}_{T_k}) {\varvec{n}}_{T_k} \end{aligned}$$

where analogously to the coil–coil collisions, $k_{w}$ denotes the coil–wall contact spring constant, $\gamma _{w}$ is the dissipation coefficient and ${\varvec{n}}_{T_k}$ stands for the outer normal vector of the triangle $T_k$.

Next we enrich our contact model by introducing friction in tangential direction. We assume only slip friction in direction of the relative tangential velocities between the edges that are in contact. The relative velocities $\dot{{\varvec{x}}}_{i,i+1} - \dot{{\varvec{x}}}_{j,j+1}$ give rise to the relative tangential velocity by

$$\begin{aligned} v_{\textrm{CC},\Vert }&=(\dot{{\varvec{x}}}_{i,i+1} - \dot{{\varvec{x}}}_{j,j+1})\nonumber \\&-\big (\dot{{\varvec{x}}}_{i,i+1} - \dot{{\varvec{x}}}_{j,j+1} \big )\cdot {\varvec{d}}^{ij}_{\textrm{min}} \frac{{\varvec{d}}^{ij}_{\textrm{min}}}{\Vert {\varvec{d}}^{ij}_{\textrm{min}}\Vert ^2} . \end{aligned}$$

**Fig. 8:**
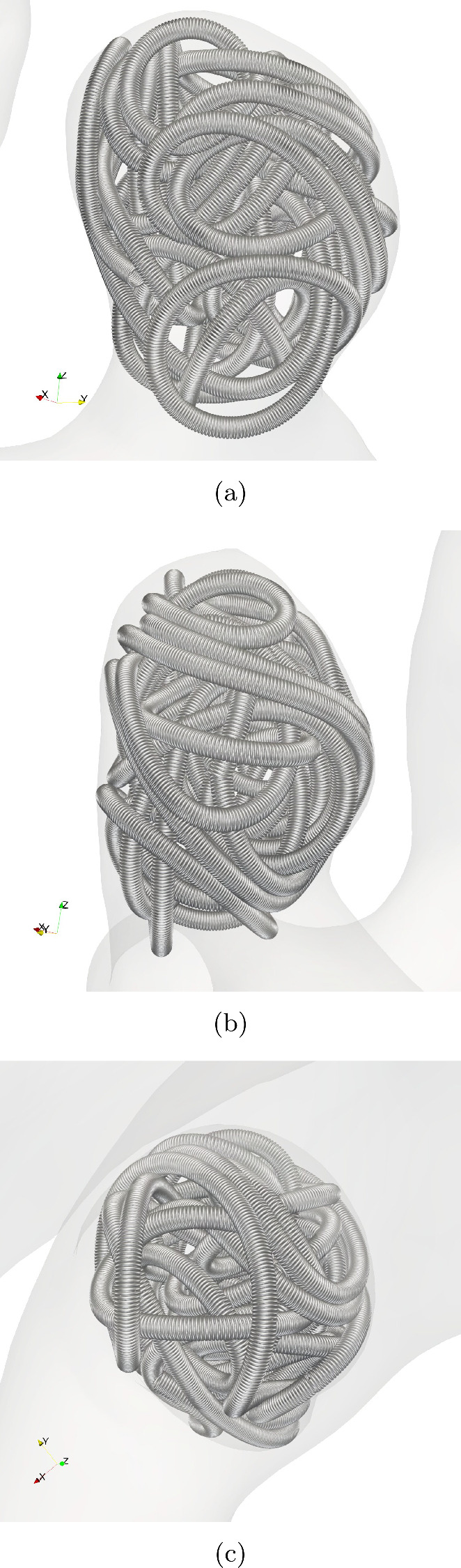
Close up view of a coil inserted into the small aneurysm at 20% global packing density. **a**, **b** side views, **c** view from bottom

Then the friction force for coil–coil contacts is

19 $$\begin{aligned} ({\varvec{F}}^i_{\hbox {CC},||})_j = -\mu _{\textrm{slip,CC}}\Vert {\varvec{F}}_{\perp }\Vert \frac{v_{\hbox {CC},\Vert }}{\Vert v_{\hbox {CC},\Vert }\Vert } \end{aligned}$$

with the slip friction coefficient $\mu _{\textrm{slip,CC}}$. Finally, the tangential friction force between wall and coil is

20 $$\begin{aligned}&\text {if } \Vert {\varvec{v}}_{\Vert }\Vert \le v_\epsilon :\nonumber \\&\left( {\varvec{F}}^i_{\hbox {CW},||}\right) _k = -\min \left\{ \Vert {\varvec{F}}_{||}\Vert , \mu _{\textrm{stick,CW}}\Vert {\varvec{F}}_{\perp }\Vert \right\} \frac{{\varvec{F}}_{||}}{\Vert {\varvec{F}}_{||}\Vert }\nonumber \\&\text {else }: \nonumber \\&\left( {\varvec{F}}^i_{\hbox {CW},||}\right) _k = -\mu _{\textrm{slip,CW}}\Vert {\varvec{F}}_{\perp }\Vert \frac{{\varvec{v}}_{\Vert }}{\Vert {\varvec{v}}_{\Vert }\Vert }, \end{aligned}$$

with $\mu _{\textrm{slip}}, \mu _{\textrm{stick}}$ denoting the coefficients of stick and slip friction, and $v_\epsilon$ being the threshold that decides when the model switches between stick and slip friction. All in all, our forces are thereby given as ${\varvec{F}}_{\textrm{ext}}={\varvec{F}}_{\hbox {CW}}+{\varvec{F}}_{\hbox {CC}}$. To conclude this section, we assume that the micro-catheter is modeled as a cylindrical surface without lids following a spline curve that is generated by 3 points in space. By triangulating its surface, we can consider it as rigid object and model its collisions with the coil in the same ways as done in (20). This concludes our discussion of the model we use of the endovascular coil embolization. An example for an embolized coil in the small aneurysm is given in Fig. 8. Here (a), (b) show side views. The view (c) is from bottom, looking through the aneurysm neck into the aneurysm.

## In silico Raymond–Roy-type occlusion classification

In this section, we propose an in silico Raymond–Roy-type classification based on the coil deployment. To do so, we follow the classical Raymond–Roy classification idea and introduce four different types of classes. The affiliation to a class is based on local packing densities (PD) at the neck and wall regions.

### Raymond–Roy-type occlusion classification

In clinical practice, the placement of a coil inside an aneurysm is of major importance. Poor placements can lead to complications such as a coil protruding into the parent artery or complications in the healing process and even aneurysm recurrence (Mascitelli et al. 2015; Kim et al. 2021).

Due to the complicated three-dimensional structure of embolized coils, a differentiation of a good and poor placement is not an easy task. For this reason, the Raymond–Roy occlusion classification (RROC) model was developed (Roy et al. 2001; Mascitelli et al. 2015). In the RROC, two different angiographic views are considered to grade an embolized coil by the following classes:

- *Class I* Complete obliteration, angiographic views from two perspectives show a complete occlusion/obliteration of the aneurysm by the coil.
- *Class II* Similar to Class I, but the aneurysm has a residual neck that is not occluded from the flow.
- *Class IIIa* Occlusion only on the aneurysm walls, but residual contrast agent shows in the core of the coil.
- *Class IIIb* Regions at the wall of the aneurysm are not correctly occluded.

From the perspective of a clinician, a coil of Class I is desired to lower the chances of an aneurysm recurrence. Note that a Class III and Class II have some probability to switch into a Class I over time, since blood clotting enhances the occlusion mechanism significantly. In Mascitelli et al. (2015), it is shown statistically that Class IIIa, compared to Class II, has a higher chance to improve to the desired Class I. Moreover, looking at the case where a complete occlusion never occurs, even after blood clotting takes place, Class IIIb, compared to Class IIIa, has a higher chance to remain incompletely occluded.

**Fig. 9:**
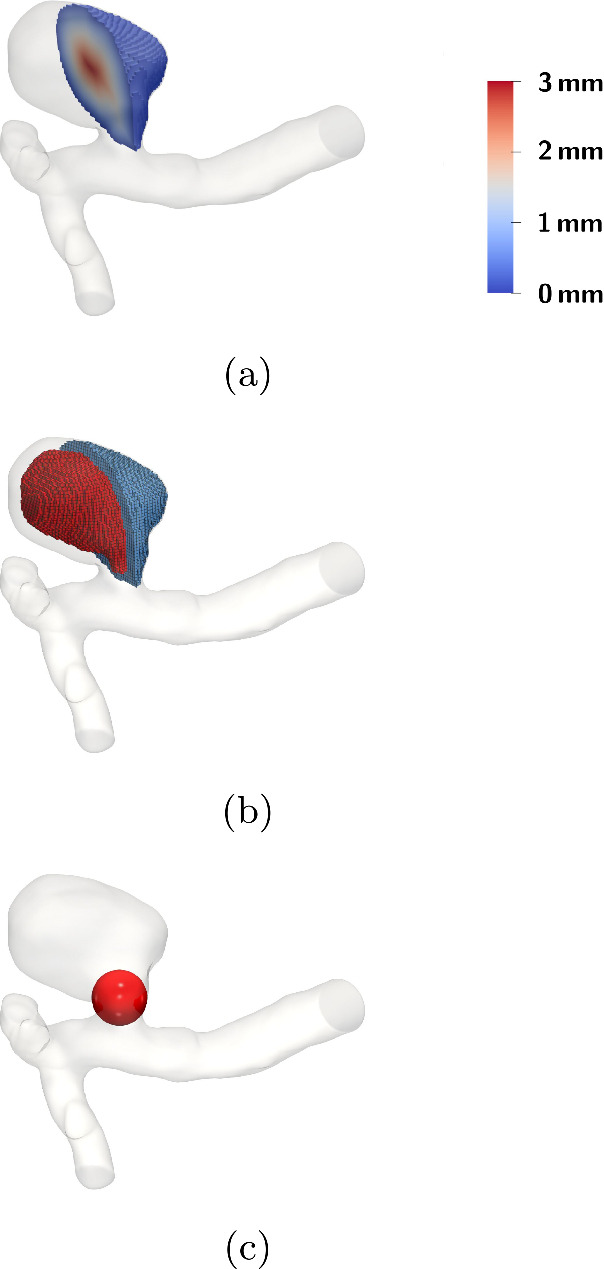
Setup for the evaluation of the RROC in case of the narrow neck aneurysm. **a** A signed distance field (Park et al. 2019) (SDF) w.r.t. the distance to the aneurysm surface is generated. **b** A level set of the SDF is used to partition the aneurysm into a boundary region (blue) and a core region (red). **c** A sphere is defined at the neck of the aneurysm to analyze the coil distribution within it

In this paper, we consider a simplified numerical version of the original RROC; namely, instead of performing an angiography, we solely analyze the local packing density of a given coil within the aneurysm (Fig. 9).

### Voxelization of coil deployments

For the RROC classification, it is convenient to convert the deployed coil, given as a set of sorted vertices ${\varvec{x}}={\varvec{x}}_0,{\varvec{x}}_1,...,{\varvec{x}}_{\textrm{n}}$ with radius $D_2$, into a more convenient form. We do this by means of voxelization, namely converting the 3D coil into a scalar field that approximates it. Without loss of generality, we set the origin of the Cartesian coordinates system such that all points within the aneurysm have nonnegative coordinates, and for each coordinate direction, there exists one point with zero component. Let $\Omega _{A}\subset {\mathbb {R}}^3$ be the space occupied by the aneurysm. Then, we can find a cube ${\mathcal {C}}_{a}$ with edge length of *a* such that $\Omega _A \subset {\mathcal {C}}_a$, meaning it is a bounding box containing the aneurysm. Next we partition the bounding box into small cubes ${\mathcal {C}}_{N_V}$ of size $a/N_V$ such that

21 $$\begin{aligned} {\mathcal {C}}_a&= \bigcup _{i,j,k=0,1, ..., N_v-1} {\mathcal {C}}_{N_V}+ \begin{bmatrix} i a/N_V \\ j a/N_V \\ k a/N_V \end{bmatrix} \nonumber \\&=\bigcup _{i,j,k=0,1, ..., N_v-1} {\mathcal {C}}_{N_V,ijk}. \end{aligned}$$

Each of the small cubes ${\mathcal {C}}_{N_V,ijk}$ has a central point $c_{N_V,ijk}=a/N_V(i+1/2,j+1/2,k+1/2)^T$. Now assume that we sweep a circle with radius $D_2$ in tangential direction of the coil centerline ${\varvec{x}}$. Then this defines a three-dimensional geometry of the coil which we call $\Omega _C$. This now allows to generate the voxelization by a binary mapping ${\mathcal {V}}:{\mathcal {C}}_a \rightarrow \{0,1\}$ with

22 $$\begin{aligned} {\varvec{y}}\in {\mathcal {C}}_a \mapsto&{\left\{ \begin{array}{ll} 1 &{} \text { if }y\in {\mathcal {C}}_{N_V,ijk} \text { and } c_{N_V,ijk} \in \Omega _C \\ 0 &{} \text {else} \end{array}\right. }\nonumber \\&\text { for } i,j,k\in \{0,...,N_V-1\}. \end{aligned}$$

**Fig. 10:**
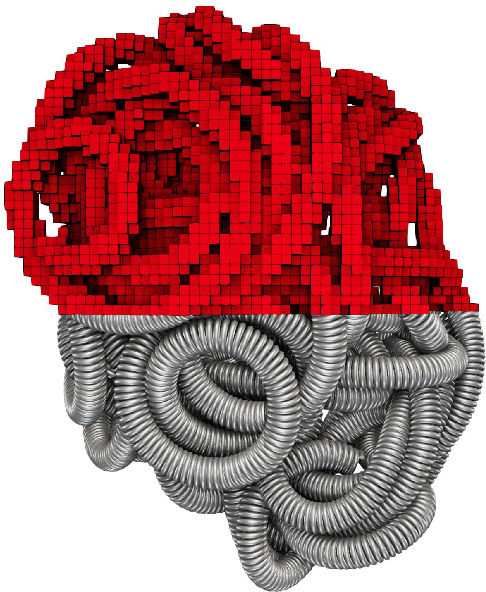
Example of a voxelization of a coil. Only voxels with value 1 (representing coil) are shown

Finally, we note that if $N_V \rightarrow \infty$ and $\Omega _A, \Omega _C$ behave sufficiently well then $\int _{{\mathcal {C}}_a}{\mathcal {V}}({\mathcal {C}}_a)~d x\rightarrow \int _{\Omega _C}1~d x$. Therefore, we need to choose $N_V$ in the definition of ${\mathcal {V}}$ large enough to represent $\Omega _C$ sufficiently well. Before concluding this section, we state how we determine whether a point ${\varvec{y}}\in {\mathcal {C}}_a$ is located inside the coil. Introducing the signed distance function (SDF) for a surface $\partial \Omega$ by

23 $$\begin{aligned} {\mathcal {F}}_{\partial \Omega }({\varvec{y}}) = {\left\{ \begin{array}{ll} \inf _{{\varvec{z}}\in \partial \Omega } -\Vert {\varvec{y}}-{\varvec{z}} \Vert &{} \text { if } {\varvec{y}}\in \Omega \\ \inf _{{\varvec{z}}\in \partial \Omega } \Vert {\varvec{y}}-{\varvec{z}} \Vert &{} \text {else} \end{array}\right. }, \end{aligned}$$

we can easily decide if a point ${\varvec{y}}$ is located inside the coil $\Omega _C$. This is exactly the case for ${\mathcal {F}}_{\partial \Omega _C}({\varvec{y}}) \le 0$. Efficient implementations to generate signed distance functions for triangulated surfaces are readily accessible, and in this work, we use the algorithm of (Park et al. 2019). An example of a voxelized coil is shown in Fig. 10. Here and thorough this work we voxelize the coil by 70 voxels in each basis vector direction of the Cartesian coordinate system. In the figure, the voxels that correspond to coil are shown in red on the top section while on the bottom the transition to the actual coil is shown. The full rectangular voxel domain can be used to compare different coil in an aneurysm if we fix the position of the voxels. The idea here is to compare between voxels that correspond to coil for different coils which we describe below in more detail.

### In silico classification

As stated before, our in silico classification is motivated by the RROC. Our classifier operates by considering the geometrical features of coil and aneurysm. For an extraction of features, we first partition the aneurysm into a core, boundary and neck region. Figure 9 shows the partitioning of the example of the narrow neck aneurysm. The partitioning is based on an SDF defined with respect to the aneurysm wall $\partial \Omega _A$, see Fig. 9a. The aneurysm is then partitioned into a core and boundary region of equal volume by choosing the level set of the SDF that generates the interface for separating these regions. In Fig. 9b, we see that the partition can be directly generated on the voxelized domain, allowing us to count the nonzero voxels ${\mathcal {V}}({\mathcal {C}}_a)$ therein and therefore obtaining the approximate coil volume in the respective regions. The neck region of the aneurysm is covered by a sphere as shown in Fig. 9c. Having the center point of the sphere, we can assess the voxels located inside the sphere and calculate the approximate coil volume contained in the sphere. Having extracted the coil volume at the boundary core and sphere region of the aneurysm enables us to construct our classifier. To fix a classification scheme, we classify a coil by Table 1.

**Table 1:** The table contains the assignment rules for the classes in our modified RROC. An aneurysm is ranked as core full when its core packing density reaches ${20\mathrm{\%}}$ and above and is ranked core empty if its core packing density is below ${20\mathrm{\%}}$. It is ranked boundary full if its boundary packing density reaches ${18\mathrm{\%}}$ and above and is ranked boundary empty if its packing density is below ${18\mathrm{\%}}$. Lastly it is ranked sphere full when the packing density in the sphere is at least ${18\mathrm{\%}}$ and sphere empty if the packing density in its sphere is below ${18\mathrm{\%}}$. We assume that the cases where we rank coil empty and boundary empty lead to coils that migrated into the parent vessel and therefore are classified as fail

Boundary full		
	Sphere full	Sphere empty
Core full	I	II
Core empty	IIIa	IIIa

Boundary empty		
	Sphere full	Sphere empty
Core full	IIIb	IIIb
Core empty	Fail	Fail

The table is motivated by the following assumptions. Having sufficient coil in the sphere blocks blood from flowing into the aneurysm. This then blocks tracer fluid from entering the aneurysm. Combined with a high packing density at the aneurysm boundary and core, no tracer fluid would be visible and Class I is assigned. If boundary and core packing densities are large enough, but the sphere is not sufficiently filled, we classify the coil as Class II, because of the possibility that tracer fluid enters the neck region. Whenever the boundary is sufficiently packed and the core is empty, we classify the coil as Class IIIa, since in this situation tracer fluid can be trapped in the core. Having a core that is sufficiently filled by an empty boundary suggests that the vessel wall is only partially occluded from the blood flow, leading to Class IIIb whether the sphere is filled or not. Finally, a case in which neither in the core nor at the boundary there is a large enough coil packing density despite the fact that a sufficiently large enough coil volume is inserted is marked as failed. In such a case, the coil has at least partially migrated into the parent artery.

## Simulation of coil placements

This section showcases the capabilities of the DER model in the context of medical coils. At first, we carry out a qualitative validation of the model. Then we test the model by applying different natural coil shapes and deploy them in the three aneurysms given in Fig. 2. The general setup for our simulation parameters is given in Appendix B.

### Inextensibility constraint

Our model approximates the inextensibility by including a penalty term in (4). Penalty approaches are easy to implement, but it is difficult to choose the penalty parameter properly. A too small parameter possibly results in a not accurate enough approximation of the constraint. A too large parameter possibly results in numerical problems, such as oscillations. To choose a suitable penalty parameter $\alpha >0$ in (4), we test the influence of $\alpha$ in the setup of Fig. 14b. For each $\alpha$ value, we calculate at each time step $t_{\textrm{n}}$ of the simulation, the relative stretch in the segments $\Delta {e}^i(t_{\textrm{n}}) = |\Vert {\varvec{e}}^i(t_{\textrm{n}}) \Vert /{\overline{\ell }} - 1|$. From this, we calculate the time averaged relative stretch as $\overline{\Delta {e}}_t^i = 1/(N_T - n_{T_0,i}) \sum _{n=n_{T_0,i}}^{N_T-1} \Delta {e}^i(t_{\textrm{n}})$ and the time variance of the relative stretch $(\overline{\Delta {e}}_{t,\sigma }^{i})^2 = 1/(N_T - n_{T_0,i}) \sum _{n=n_{T_0,i}}^{N_T-1}{\big ( \overline{\Delta {e}}_t^i - \Delta {e}^i(t_{\textrm{n}})\big )^2}$. By the index $n_{T_0,i} \in \{0,1,...,N_T-1\}$, we denote the time step at which node ${\varvec{x}}_i$ is pushed out of the micro-catheter, while $N_T$ stands for the total number of time steps. We use the following three quantities to analyze the relative degree by which the penalty is satisfied

24 $$\begin{aligned} \overline{\Delta {e}}&= \frac{1}{N-1}\sum _{i=0}^{N-2} \overline{\Delta {e}}_t ^i, \nonumber \\ \overline{\Delta {e}}_{\sigma }&= \frac{1}{N-1} \sum _{i=0}^{N-2} \overline{\Delta {e}}_{t\sigma }^i,\nonumber \\ \Delta L&= \frac{1}{L}\bigg | L-\sum _{i=0}^{N-2} \Vert {\varvec{e}}^i(t_{N_T-1})\Vert \bigg |. \end{aligned}$$

Here, $N-1$ is the number of edges created by the *N* nodes on the discrete coil centerline. Note that $\overline{\Delta {e}}$ and $\overline{\Delta {e}}_{\sigma }$ are the time averaged relative stretch and the time variance of the relative stretch averaged over all edges, while $\Delta L$ is the relative stretch of the whole coil.

**Table 2:** Influence of the penalty parameter $\alpha$ on $\overline{\Delta {e}}$ as the mean relative stretch averaged over time and coil length, $\overline{\Delta {e}}_{\sigma }$ as the standard deviation of the time averaged relative stretch with respect to time and averaged over the coil length. $\Delta L$ the difference of the rod length in its final configuration relative to its initial length

$\alpha$	$\overline{\Delta {\varvec{e}}}$ in %	$\overline{\Delta {\varvec{e}}}_{\sigma }$ in %	$\Delta L$ in %
0.025	1.58	19.47	0.20
0.05	0.83	10.55	0.124
0.1	0.52	5.1	0.053
0.2	0.20	2.98	0.03

In Table 2, the results of this numerical study are summarized. We conclude that with $\alpha =0.1$, the penalty is sufficiently effective, since the local relative segment extension is below ${1\mathrm{\%}}$ with a standard deviation of approximately ${5\mathrm{\%}}$ and the total relative extension is below ${0.1\mathrm{\%}}$.

### Range of forces

We have also checked whether the magnitude of the bending and torsion forces lie in a realistic range. Comparing it to the experiment in Okuda et al. (2022), we found that averaging the bending force over time and taking the maximum value over the coil length results in a magnitude of about ${4e-3\,\mathrm{\text {N}}}$, while the force due to twist is much lower. In the experiment, the total forces of the coil on a synthetic aneurysm wall were measured and are approximately ${1e-2\,\mathrm{\text {N}}}$. Since multiple coil loops press against the wall of the aneurysm in the experiment, we see that the computed forces are in a physical realistic range.

### Qualitative validation of model correctness

**Fig. 11:**
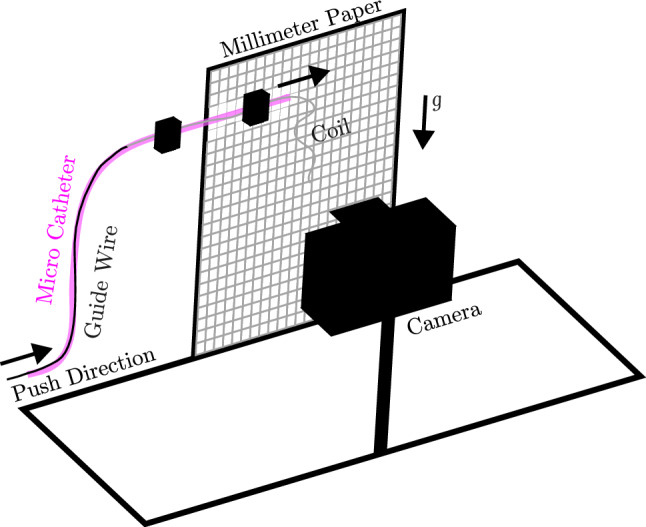
Sketch of the experimental setup

In this subsection, we give an overview of the capabilities of our model by displaying exemplary coil placements. To this end, we start by providing a qualitative validation of the model. In the validation experiment, we fix a micro-catheter in front of a millimeter paper, see Fig. 11. The micro-catheter is secured by two clamps in front of a sheet of millimeter paper in a manner that prevents it from making contact. Then by advancing the guide wire, the coil is pushed out of the micro-catheter while its shape, influenced by the gravitational acceleration *g*, is captured by the camera. In a second step, we recreate this experiment virtually by our coil model where an additional gravitational force term is included.

For the experiments, we use two platinum coils, each differing in shape and characteristic sizes.^3^ The first coil exhibits a helical shape with total length of $L={4\,\mathrm{\text {c}\text {m}}}$ and the diameters $D_2 = {510\,\mathrm{\upmu \text {m}}}$, $D_3 = {4\,\mathrm{\text {m}\text {m}}}$. The second coil shows a more complex shape and a total length of $L={45\,\mathrm{\text {c}\text {m}}}$ and the diameters $D_2 = {340\,\mathrm{\upmu \text {m}}}$, $D_3 = {10\,\mathrm{\text {m}\text {m}}}$. To ensure that the simulated coils match the experimental ones as good as possible, these parameters were directly incorporated into the numerical simulations. Parameters that are not given by the manufacturer need to be specified synthetically. To do so, we inversely estimate the unknown parameter $D_1$ by adjusting the hanging length of the coil in the simulation until it approximately matches the experiment. For both our coils, we use the given values for $L, D_2$ and $D_3$. In the helix coil simulations, we estimate that the distance between loops of the helix curve is $1.2 D_2$ and set $D_1 = {49\,\mathrm{\upmu \text {m}}}$. For the coil with a complex shape, we assume the natural shape to be as shown in Fig. 13, motivated by the patent for stable coil designs in Wallace et al. (2001). We further set $D_1 = {74\,\mathrm{\upmu \text {m}}}$.

**Fig. 12:**
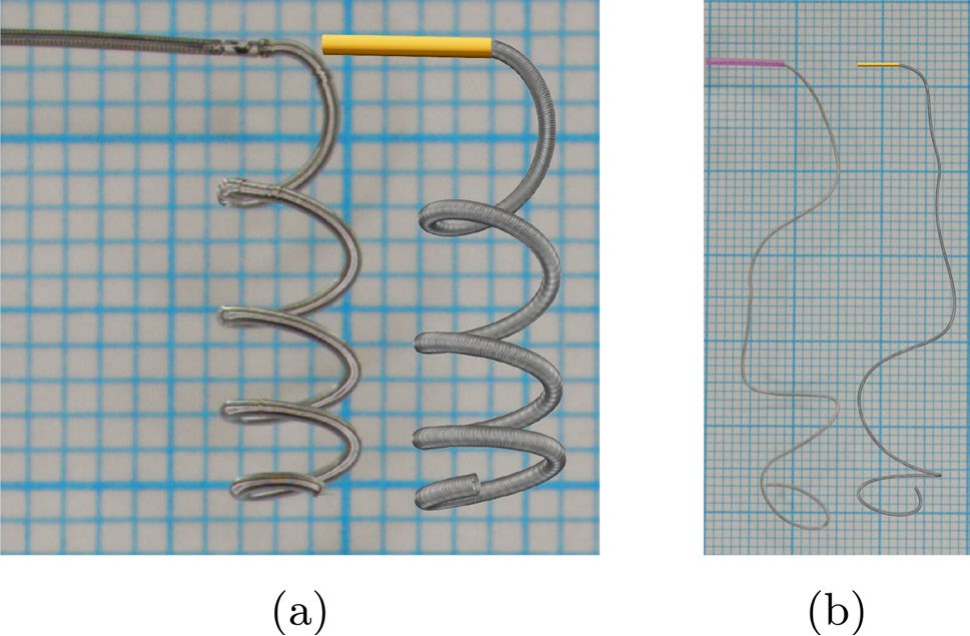
Qualitative validation of the mathematical model: coil deformation under the influence of gravitation. **a** shows a helix coil while **b** is a three-dimensional framing coil. Simulations are placed on the right besides the experiments on the left within each subfigure

For the case of the helix coil in Fig. 12a, one can see the experiment on the left and the simulation on the right. Since this helix coil is relatively short with a total length of ${4\,\mathrm{\text {c}\text {m}}}$, we have pushed it completely out of the micro-catheter such that it is only fixed to the delivery wire via the detachment mechanism. Although the position of the helix loops is slightly shifted, the overall shape matches quite well with our simulation. Due to the unknown pitch of the helix, the total length of the coil was rescaled to ${4.6\,\mathrm{\text {c}\text {m}}}$ to obtain the shown shape.

The results for the considered three-dimensional coil are shown in Fig. 12b. Here ${10\,\mathrm{\text {c}\text {m}}}$ are pushed out of the micro-catheter. Comparing the experiment on the left to the simulation on the right, we see that even though the shape is unknown the main features, e.g., the loop at the tip and the intermediate bending, are represented in the simulation.

**Fig. 13:**
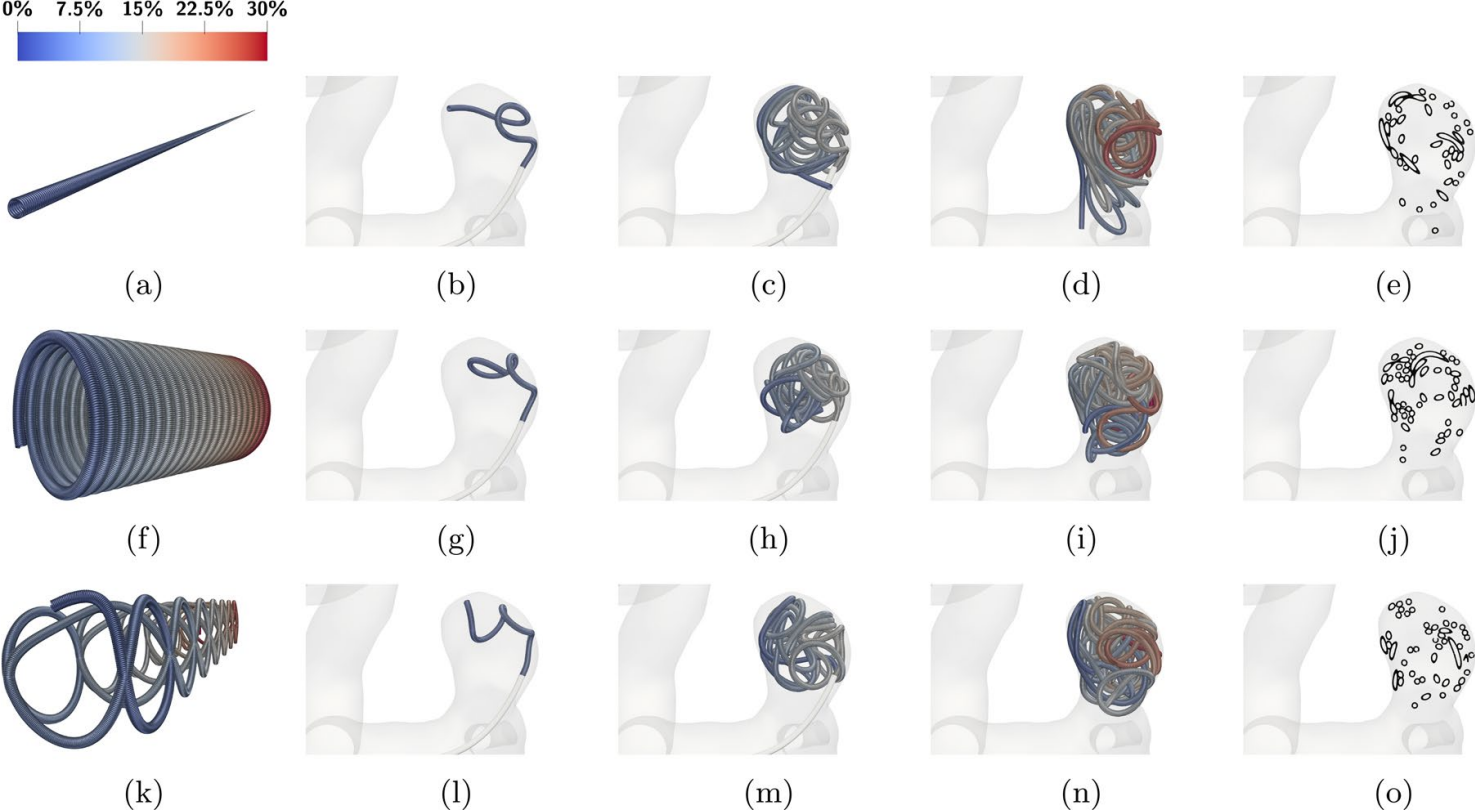
Insertion of coils with different natural curvature into the small aneurysm. The colors correspond to the coils relative arc length from 0 to 1. The coils are inserted until a packing density of $25\%$ is reached. The diameters are chosen as: $D_1={50\,\mathrm{\upmu \text {m}}}$, $D_2={305\,\mathrm{\upmu \text {m}}}$, $D_3={2\,\mathrm{\text {m}\text {m}}}$. Rows **a**–**e** correspond to the straight coil, rows **f**–**j** to the helical coil and rows **k**–**o** to the 3D shaped coil. **a**–**k** (vertically) show the natural shapes of the three coils considered. The three center columns show the insertion of each coil when ${5\mathrm{\%}}$, ${50\mathrm{\%}}$ and ${100\mathrm{\%}}$ of the embolization process is completed. In **e**–**o** (again vertically), one can see a cross section of the fully inserted coil

### Exemplary study of coil placements

In this section, we study virtual coil placements by means of our numerical method illustrating its flexibility and robustness with respect to natural shapes and aneurysm geometries.

#### Influence of the natural shape

We begin by simulating the embolization of coils with different shapes into the small aneurysm (see Fig. 2 (top)). We use shapes that are a straight, a helix pre-shaped and a 3D coil motivated by White et al. (2008); see Fig. 13. The point of insertion can be seen on the right of each picture at the tip of the micro-catheter.

The coils are chosen such that a $25\%$ global packing density is reached in the aneurysm (see red zone in Fig. 2). We observe that a straight coil leads to a placement where the coil is protruding into the artery. For the helix and 3D coils, this is not the case. Their $D_3$ diameter is set to ${2\,\mathrm{\text {m}\text {m}}}$ which is smaller than the aneurysm’s diameter (compare to Fig. 2) leading to a more uniformly distributed and compact placement. In terms of their cross-sectional distribution, we can see that the distribution of the coil at the neck is the highest for the 3D coil, whereas for the straight and helix coil there are still openings at the neck.

#### Influence of the geometry

**Fig. 14:**
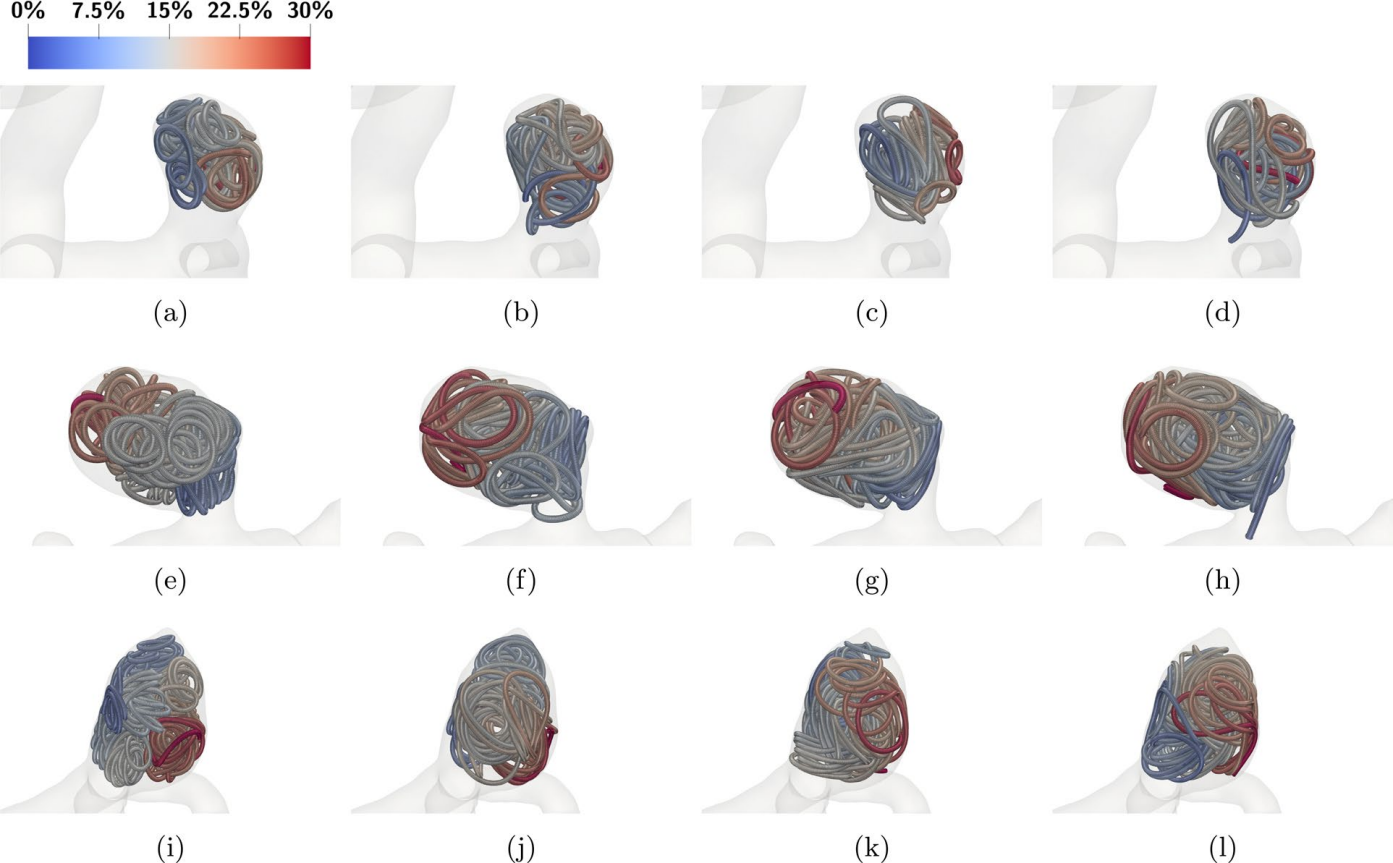
Insertion of helical coils into the different aneurysm geometries. The colors correspond to the coils relative arc length from 0 to 1. Rows a–d correspond to the small aneurysm geometry, e–h the narrow neck aneurysm geometry and i–l to the bifurcation aneurysm geometry. The coils are inserted until a packing density of $25\%$ is reached. The diameters are chosen as: $D_1={50\,\mathrm{\upmu \text {m}}}$, $D_2={305\,\mathrm{\upmu \text {m}}},$ and $D_3$ is chosen from left to right as ${2\,\mathrm{\text {m}\text {m}}}, {4\,\mathrm{\text {m}\text {m}}}, {6\,\mathrm{\text {m}\text {m}}},$ and ${8\,\mathrm{\text {m}\text {m}}}$

Next we show how the different aneurysm geometries (see again Fig. 2) affect the placement of coils. In Fig. 14 from top to bottom, the three aneurysm geometries are shown while from left to right, we set the $D_3$ diameter of the helix coil to ${2\,\mathrm{\text {m}\text {m}}}, {4\,\mathrm{\text {m}\text {m}}}, {6\,\mathrm{\text {m}\text {m}}}$ and ${8\,\mathrm{\text {m}\text {m}}}$.

In case of the small aneurysm which can be seen in the top sequence, a smaller $D_3$ radius reduces the probability of the coil protruding into the parent vessel. The three cases on the right show that a higher $D_3$ value increases the probability for protrusion. In the middle case, the neck of the aneurysm is much smaller compared to its dome diameter making it a case that can be coiled more easily. Looking at the variations of $D_3$, we can see that up to a $D_3$ diameter of ${8\,\mathrm{\text {m}\text {m}}}$ there is no protrusion into the main artery. For $D_3 ={2\,\textrm{mm}}$, high local packing densities are possible leading to a poorly (w.r.t. uniformity) distributed coil close to the walls of the aneurysm. Finally, for the bifurcating aneurysm that is located in the bottom row of Fig. 14, one can see that in all cases, the coil reaches into the main vessel. The neck of this aneurysm is relatively large, and thus, it is more difficult to insert a coil without protrusion. We note that for such geometries often a combination of a coil and a flow diverter is used. Setting $D_3 ={6\,\mathrm{\text {m}\text {m}}}$ yields the best placement, meaning that for this case the protrusion into the parent vessel is not excessive.

#### Multiple coils

In clinical applications, it is standard to place several coils within an aneurysm. This allows to firstly stabilize the geometry by a framing coil and secondly to occlude the aneurysm more densely by one or more filling coils.

**Fig. 15:**
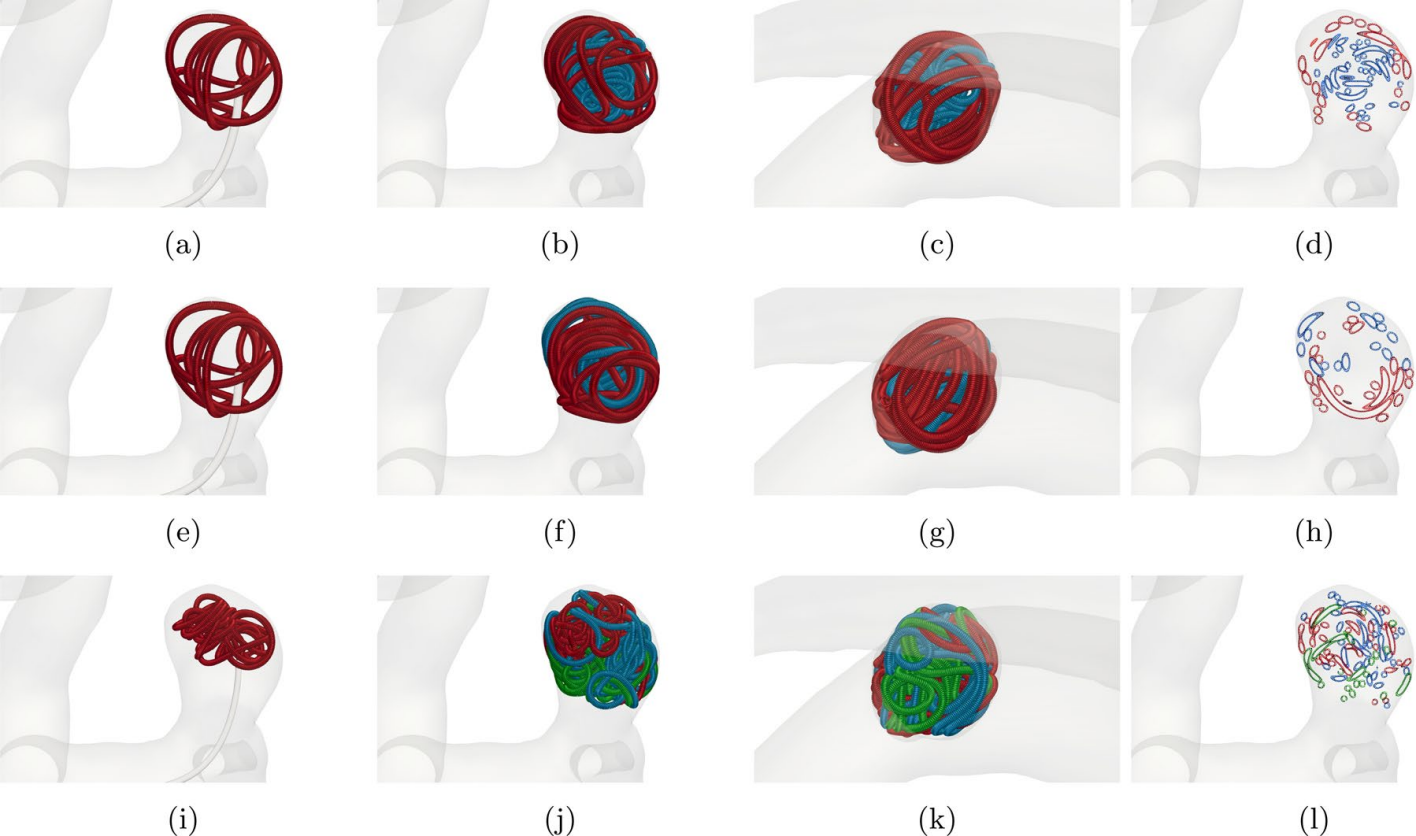
Coiling of the small aneurysm by multiple coils with PD of approximately 32%. The following two types of coils are used: 3D shape framing coil with $D_1={76\,\mathrm{\upmu \text {m}}}$, $D_2={355\,\mathrm{\upmu \text {m}}}$, $D_3={5\,\mathrm{\text {m}\text {m}}}$ and a helical filling coil with $D_1={50\,\mathrm{\upmu \text {m}}}$, $D_2={254\,\mathrm{\upmu \text {m}}}$, $D_3={2\,\mathrm{\text {m}\text {m}}}$. In the rows a–d, a framing coil (red) with length ${15\,\mathrm{\text {c}\text {m}}}$ and a filling coil (blue) with length ${20\,\mathrm{\text {c}\text {m}}}$ is inserted. Rows e–h depict the insertion of a framing coil (red) with length ${15\,\mathrm{\text {c}\text {m}}}$ and a second framing coil (blue) with length ${10\,\mathrm{\text {c}\text {m}}}$. In the last rows i–l, three filling coils (red, blue and green) are inserted. From those the first two have the length 20 cm and the last one 10 cm. The columns **a**–**i** show the insertion processes in all three coil embolizations, when the first coil is half-deployed. In **b**–**j**, the final configuration is shown, after all coils are placed. Columns **c**–**k** show the final configuration from an interior viewpoint, looking into the neck of the aneurysm while columns **d**-**l** present the coil distribution in a centralized cross section

Our model is flexible enough to be easily extended to such situations. More precisely, Eq. (12a) can be simulated for several coils by updating their positions and forces sequentially at each time step. Similarly, coil–wall collisions are handled. For the coil–coil collisions, we *virtually* connect the last node of each coil to the first node of the coil that is inserted next, effectively creating one long coil. By not considering the connecting *virtual* edges between the coils, we can handle coil–coil collisions by the same approach as in Sect. 2.6. Note that we assume that coils are pushed sequentially into the aneurysm. This section analyzes the models’ behavior when using more than one coil for occlusion. In this way, the model allows us to estimate the coil placement in a practical situation.

In Fig. 15, we have coiled the small aneurysm with multiple coils that are typically used by interventionalists. These include framing and filling coils. For more details, see Fig. 15. In each row, different types of coils are combined to occlude the aneurysm. To enable comparability between the results, the micro-catheter is in all cases fixed, and the final PD is approximately 32%. Figure 15, rows (a)–(d), shows the clinically widely used combination of a filling coil and a framing coil. Rows (e)–(h) show occlusion by two framing coils and rows (i)–(l) with three filling coils. Considering the left column in Fig. 15, one can see in (a) and (e) that the first framing coil forms a stable cage inside the aneurysm, while the first filling coil in (i) does not expand to the walls and is less mechanically secured. The final placement after inserting the remaining coils is shown in columns (b)–(j). The situation of inserting a framing coil followed by a filling coil yields a robust and yet quite uniformly packed aneurysm sack, see (b). Having two framing coils inserted gives a very stiff cage but almost no filling at the aneurysm sack center, see (f). The most uniformly packed aneurysm sack is obtained by the insertion of three filling coils as shown in (j). The neck occlusion after insertion of all coils is shown in columns (c)–(k) from an inferior view. In the two cases that make use of framing coils (c) and (g), it can be seen that these can prevent the remaining coil loops in the aneurysm from migration into the parent artery. The filling coil (k) has in the neck region several smaller loops which can bear together with its lower stiffness the risk of migration. The final cross section in (d), (h) reveals that the distribution of coil is most even when inserting the three filling coils (h). For the insertion of two framing coils the coverage of the aneurysm center is relatively low. Compared to that, framing and filling coil in (d) covers the center region better than (h) but arguably less uniform than (l).

From this study, we conclude that for the consecutive insertion of the selected framing and filling coil, a mechanically stable coil with even distribution in the aneurysm center is formed. For the two consecutive inserted filling coils, the coil distribution in the center of the aneurysm might not be sufficient for a proper clotting response. In case of with three filling coils, the coil distribution is most even but can migrate in the parent artery. In this case, a flow diverter for additional support can be used.

For the remainder of the paper, we restrict ourselves to the placement of only one coil in the aneurysm. Understanding single coil procedures is highly relevant, when considering the impact of certain design choices such as coil stiffness and -shape. We also note that smaller aneurysms, which are the most frequent ones, can indeed be occluded by one coil (Vanzin et al. 2012). Moreover, a structured analysis of all clinical relevant coil combinations results in a high-dimensional problem. A rigorous sensitivity study on such a high-dimensional parameter space goes beyond the scope of this paper.

### Analyzing coil distributions

To assess grades of occlusion by the RROC, we proceed as in Fig. 9. First a SDF is generated. The SDF enables a natural partitioning of the aneurysm by its level sets. In our case, we choose the level set in such a way that the aneurysm core and boundary region are two partitions of equal volume (see Fig. 9b blue and red region). Having the coil distribution in the boundary region enables us to judge if a coil belongs to the Class IIIb or vice versa the filling of the core region which—if insufficient—leads to a Class IIIa grade. Finally, to assess Class II, we analyze the volume fraction in a sphere at the neck of the aneurysm (see Fig. 9c).

To actually calculate the volume fractions, we use the voxelization for each coil as discussed in Sect. 3.2. To analyze the sensitivity of our numerical coil model with respect to the simulation parameters, we sample the simulation parameters, i.e., the Young’s modulus, from a distribution $Z \sim {\mathcal {D}}$ and generate the respective coil placements with their voxelization $\psi (Z)$. Those can then be used to calculate the empirical mean and standard deviation of the coil distribution via

25 $$\begin{aligned} {\overline{\psi }}&= \frac{1}{N}\sum \limits _{i=1}^N \psi (Z_{i}), \nonumber \\ \psi _{\sigma }^2&= \frac{1}{N}\sum \limits _{i=1}^N \left( {\overline{\psi }}-\psi (Z_{i})\right) ^2 . \end{aligned}$$

Note that we assume that the voxelized coil distributions $\psi$ are defined on the same domain and therefore can be summed up in the notation above in a point-wise manner. From this, we calculate the volume of a mean coil distribution in a subregion of our aneurysm domain *V* and its standard deviation with respect to the voxelized coil distribution $\psi (Z_{i})$ by

26 $$\begin{aligned} {\overline{\psi }}_V&=\int _{V} {\overline{\psi }}~{\text{d}}x, \nonumber \\ \psi _{\sigma , V}^2&=\frac{1}{N}\sum \limits _{i=1}^N\bigg (\int _{V} {\overline{\psi }}-\psi (Z_{i})~{\text{d}}x\bigg )^2 \end{aligned}$$

which enables us to generate a volume fraction ${\overline{\psi }}_V/{\widetilde{V}}$ with respect to a reference volume ${\widetilde{V}}$. For the core, boundary and sphere region in the aneurysm (see Fig. 9), we compute the following volume fractions

27 $$\begin{aligned} {\overline{\psi }}_{\textrm{BA}}=\frac{{\overline{\psi }}_{V_\text {B}}}{V_\text {A}}, {\overline{\psi }}_{\textrm{CA}}=\frac{{\overline{\psi }}_{V_\text {C}}}{V_\text {A}}, {\overline{\psi }}_{\textrm{AA}}=\frac{{\overline{\psi }}_{V_\text {A}}}{V_\text {A}}, {\overline{\psi }}_{\textrm{SS}}=\frac{{\overline{\psi }}_{V_\text {S}}}{V_\text {S}} \end{aligned}$$

where $V_{\text {C}}$, $V_{\text {B}}$, $V_{\text {A}}$ and $V_{\text {S}}$ are the volumes of the core, boundary and full aneurysm and the sphere with $V_{\text {A}}=V_{\text {B}}+V_{\text {C}}$. Note that in $V_{\text {S}}$, only the volume of the sphere within the aneurysm is considered.

**Fig. 16:**
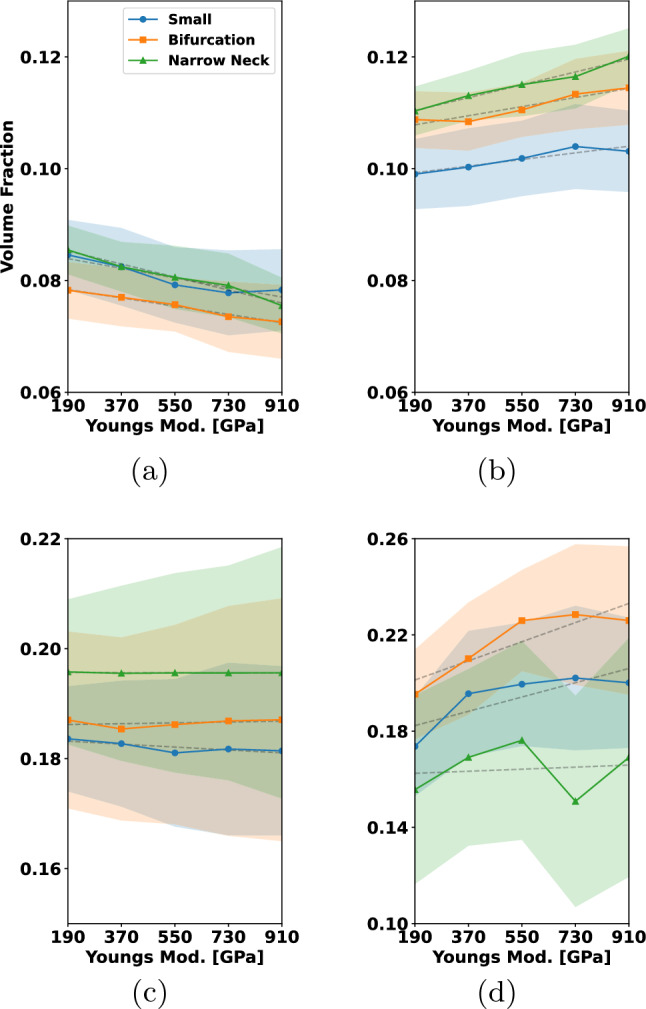
Variation of Young’s modulus helix coil: **a** volume fraction of coil in aneurysm boundary, **b** volume fraction of coil in aneurysm core, **c** total volume fraction of coil in aneurysm, **d** volume fraction of coil in the sphere at the aneurysm neck

In the following, we study the influence of the main variables $E_{w}, D_2, D_3$ of the coil on the volume fractions (27). To this end, we proceed in the following way:

1. Let *Z* be a random variable corresponding to $E_{\text {w}}, D_2, D_3$ and sample it uniformly $Z\sim {\mathcal {U}}[I(Z)]$ within the interval *I*(*Z*).
2. For each sample $Z_i$ simulate the coil under fixed parameters and generate the voxelization $\psi (Z_i)$.
3. Create a partition of *I*(*Z*) by 5 subintervals $I_1(Z)\cup \cdots \cup I_5(Z)$.
4. On each subinterval, we compute ${\overline{\psi }}$ by (25) integrate it on a volume region ${\widetilde{V}}$(26) and calculate the volume fractions in (27).

**Fig. 17:**
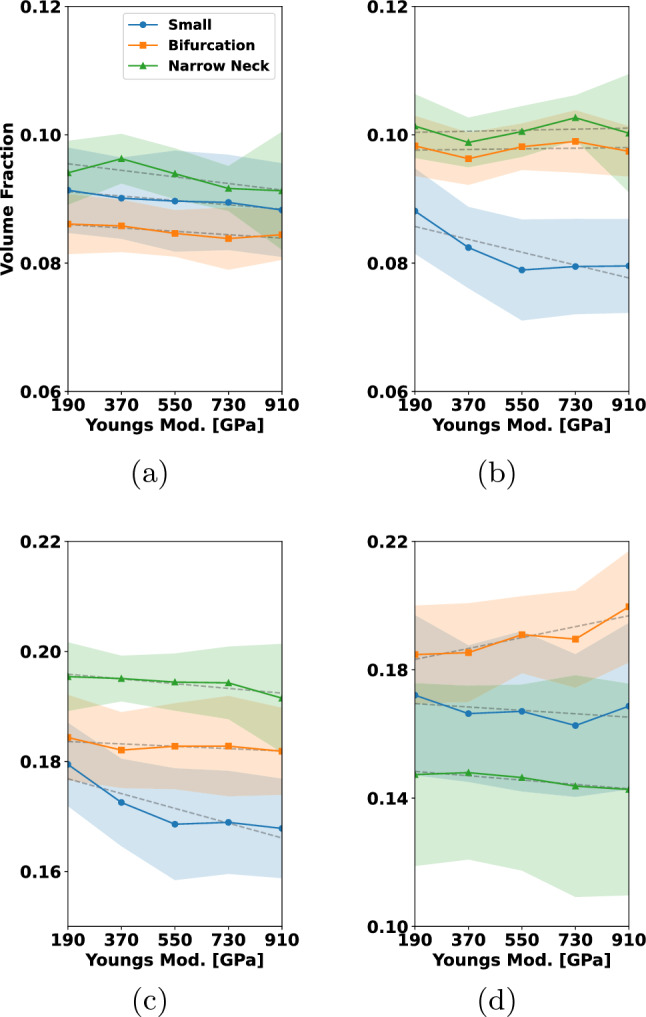
Variation of Young’s modulus 3D coil: **a** volume fraction of coil in aneurysm boundary, **b** volume fraction of coil in aneurysm core, **c** total volume fraction of coil in aneurysm, **d** volume fraction of coil in the sphere at the aneurysm neck

**Fig. 18:**
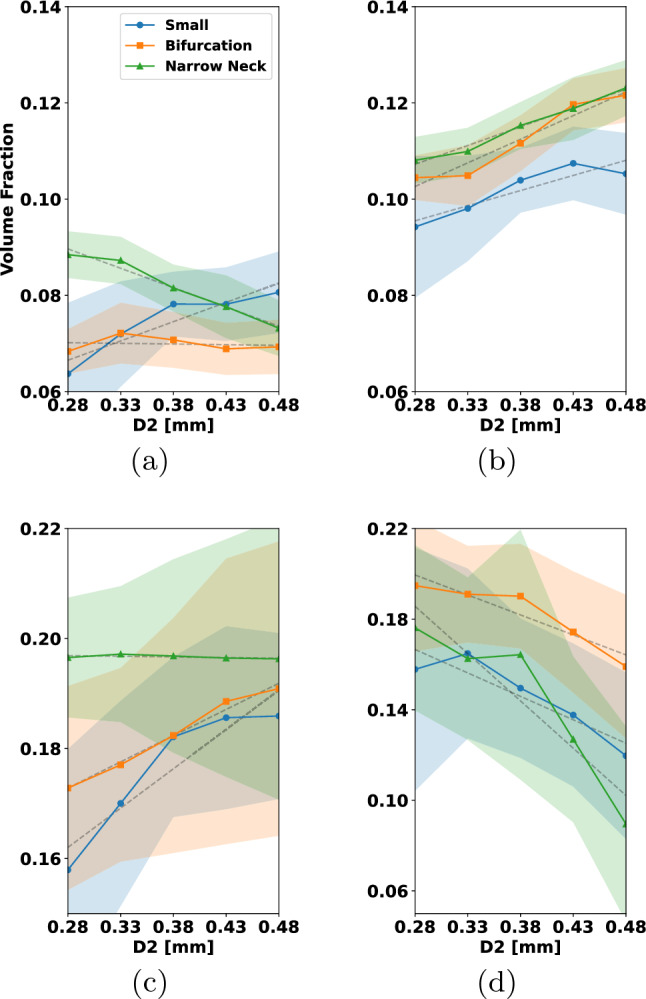
Variation of $D_2$ helix coil: **a** volume fraction of coil in aneurysm boundary, **b** volume fraction of coil in aneurysm core, **c** total volume fraction of coil in aneurysm, **d** volume fraction of coil in the sphere at the aneurysm neck

**Fig. 19:**
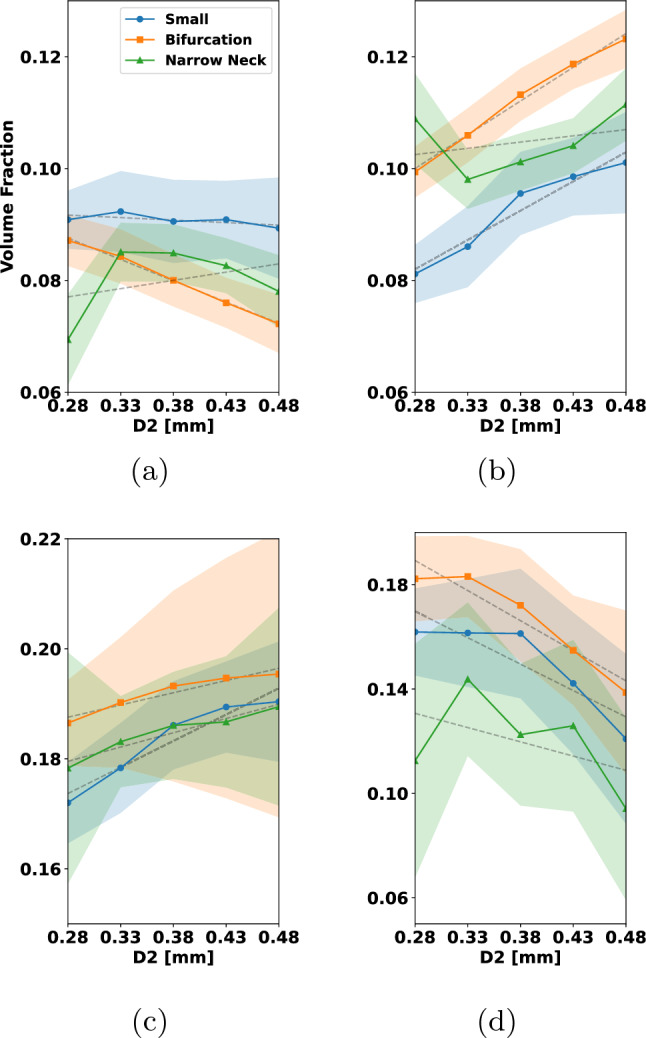
Variation of $D_2$ 3D coil: **a** volume fraction of coil in aneurysm boundary, **b** volume fraction of coil in aneurysm core, **c** total volume fraction of coil in aneurysm, **d** volume fraction of coil in the sphere at the aneurysm neck

**Fig. 20:**
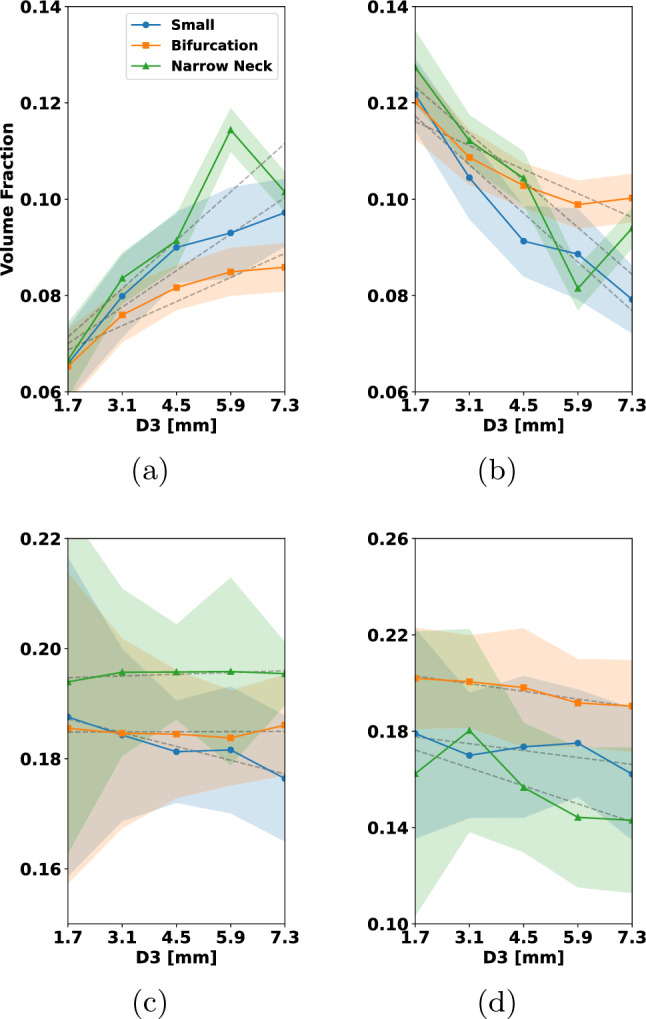
Variation of $D_3$ helix coil: **a** volume fraction of coil in aneurysm boundary, **b** volume fraction of coil in aneurysm core, **c** total volume fraction of coil in aneurysm, **d** volume fraction of coil in the sphere at the aneurysm neck

In order to compute averages and confidence intervals reliably, we ensure that each subinterval contains at least 30 samples of *Z*. Each of the cases is simulated with a final packing density of 20%. For the characteristic lengths, we set $D_1$ to 50 $\upmu$m, $D_2$ to 500 $\upmu$m, and $D_3$ to 4 mm when not otherwise stated. We depict our findings in Figs. 16, 17, 18, 19 and 20. The plots show on the abscissa the center of the 5 intervals $I_1(Z),...,I_5(Z)$ and on the ordinate the averaged volume fraction on the corresponding interval. The figures are sorted such that ${\overline{\psi }}_{\textrm{BA}}$ corresponds to (a), ${\overline{\psi }}_{\textrm{CA}}$ is (b), ${\overline{\psi }}_{\textrm{AA}}$ is (c) and ${\overline{\psi }}_{\textrm{SS}}$ can be found in (d). Colors correspond to the aneurysm, with blue being the small, orange being the bifurcation and green being the narrow neck aneurysm. Confidence intervals are given in the same colors as faded areas in the background.

We start by showing the influence of a variation in the Young’s modulus on the volume fractions with $E_{w}\sim {\mathcal {U}}[{1e2\,\mathrm{\text {G}\text {Pa}}},{1e3\,\mathrm{\text {G}\text {Pa}}}]$.

*Variation of Young’s modulus for a helix coil * Fig.16: For ${\overline{\psi }}_{\textrm{BA}}$ in (a) a slight decrease can be observed when $E_{{w}}$ is increased. This leads to an increase in ${\overline{\psi }}_{\textrm{CA}}$ in (b) when $E_{{w}}$ is increased. Considering ${\overline{\psi }}_{\textrm{AA}}$ in (c) the volume fraction is relative constant for all three aneurysms. The highest value is observed in the narrow neck aneurysm and the lowest in the small aneurysm. Looking at ${\overline{\psi }}_{\textrm{SS}}$ in (d), we can see that the volume in the sphere increases for larger $E_{{w}}$ but stagnates when reaching about 550 GPa.

*Variation of Young’s modulus for a 3D coil * Fig.17: In this case, ${\overline{\psi }}_{\textrm{BA}}$ is relatively constant when changing $E_{{w}}$ in (a). For ${\overline{\psi }}_{\textrm{CA}}$ in (b), we observe for the small aneurysm a downwards trend when increasing $E_{{w}}$ to a value of ${550\,\mathrm{\text {G}\text {Pa}}}$. The same trend of the decrease in the volume fraction for the small aneurysm is visible in ${\overline{\psi }}_{\textrm{AA}}$ while it is relatively constant for the other two geometries. Lastly we note that there is a slight increase in ${\overline{\psi }}_{\textrm{SS}}$ when increasing $E_{{w}}$ which is approximately constant in the other geometries.

We summarize that the variation of $E_{{w}}$ has a greater impact on the helix coil where it causes the coil to migrate from the boundary region into the core region. Moreover, increasing $E_{{w}}$ leads to an increase in coil volume at the neck when a helix coil is used. The helix coil is more densely packed in the core region while the 3D coil is more densely packed in the boundary region. For the helix coil, the packing in the neck region is higher than in the 3D coil case as one can see by comparing the results in (d).

Next we focus on the variation of $D_2$ where the sampling interval is chosen as $D_2\sim {\mathcal {U}}[{0.255\,\mathrm{\text {m}\text {m}}},{0.505\,\mathrm{\text {m}\text {m}}}]$.

*Variation of the* $D_2$ *diameter for a helix coil* Fig.18: Increasing $D_2$ leads to an increase in ${\overline{\psi }}_{\textrm{BA}}$ as can be seen in (a) for coils pushed into the small aneurysm while a decrease in this volume fraction is caused in case of the narrow neck aneurysm. For the bifurcation aneurysm there is no clear trend visible. Considering ${\overline{\psi }}_{\textrm{CA}}$, an increase in volume fraction is observed when increasing $D_2$. The volume fraction ${\overline{\psi }}_{\textrm{AA}}$ increases by approximately 3% in the small aneurysm and by 2% in the bifurcation aneurysm. For the narrow neck aneurysm, it is nearly constant. Analyzing ${\overline{\psi }}_{\textrm{SS}}$ in (d) one can see that the volume fraction of coil in the sphere is significantly impacted by $D_2$. When the coil radius is increased, the volume fraction in the sphere is lowered by up to 3% in each of the aneurysms.

*Variation of the* $D_2$ *diameter for a 3D coil* Fig.19: For the 3D coil a increase of $D_2$ lead to a decrease in ${\overline{\psi }}_{\textrm{BA}}$ for the small aneurysm and narrow neck aneurysm. Note that the lowest values of $D_2$ caused the smallest volume fraction in the core region for the narrow neck aneurysm. The impact of $D_2$ on ${\overline{\psi }}_{\textrm{BA}}$ in the small aneurysm is not significant. In (b), one can observe that the core volume fraction depends on $D_2$ and rises as $D_2$ is increased. Additionally, a small $D_2$ in (d) leads to a decrease of roughly ${2\mathrm{\%}}$ in the volume fractions of the coil within the aneurysm when compared to the volume fractions at the highest $D_2$ value. For the volume fraction in the sphere region, a higher radius $D_2$ decreases the volume fraction in the sphere region when considering the bifurcation and small aneurysm case. For the narrow neck aneurysm, a trend is less clearly visible. Concluding our findings for the variation in $D_2$, we note that $D_2$ has a substantial influence on all volume fractions. For both the helix and the 3D coil, an increase in $D_2$ lead to an overall decrease in the volume fraction ${\overline{\psi }}_{\textrm{BA}}$ in the narrow neck aneurysm. In case of the small aneurysm packed with a helix coil, a small $D_2$ value causes a lower volume fraction in the boundary region. For the bifurcation aneurysm, a 3D coil results in a smaller volume fraction at the boundary when compared to the helix coil. In the core region, an increase in $D_2$ leads to an increase in ${\overline{\psi }}_{\textrm{CA}}$. Additionally, we observe that a smaller value of $D_2$ leads to possible coil migrating into the parent vessel direction and results in large variations in sphere and total aneurysm volume. Finally, we see for both coils that a higher volume fraction ${\overline{\psi }}_{\textrm{SS}}$ is obtained by a smaller $D_2$.

Finally, we sample the diameter $D_3$ according to $D_3\sim {\mathcal {U}}[{2\,\mathrm{\text {m}\text {m}}},{8\,\mathrm{\text {m}\text {m}}}]$.

*Variation of* $D_3$ *for a helix coil* Fig.20: For each of the geometries, the volume fraction ${\overline{\psi }}_{\textrm{BA}}$ increases roughly by ${2\mathrm{\%}}$ in (a) and decreases when considering ${\overline{\psi }}_{\textrm{CA}}$ in (b). At a $D_3$ diameter of 5.7 mm–6.1 mm for the narrow neck aneurysm a peak is visible increasing ${\overline{\psi }}_{\textrm{BA}}$ by another 2%. Note that the confidence interval at the peak is smaller than 1%, meaning that it occurs for most of the samples. Looking at ${\overline{\psi }}_{\textrm{AA}}$ in (c), one can see that the coil volume fraction in the interior of the small aneurysm decreases when increasing $D_3$. Finally, we note that an increase of $D_3$ in (d) leads to a slight decrease of ${\overline{\psi }}_{\textrm{SS}}$ in all geometries. Therefore, changing $D_3$ can impact the volume of the coil at the boundary and core significantly. This behavior can be observed for framing coils where larger $D_3$ lead to coils that are more present in the boundary region of the aneurysm creating a stabilizing cage for the filling coils.

**Fig. 21:**
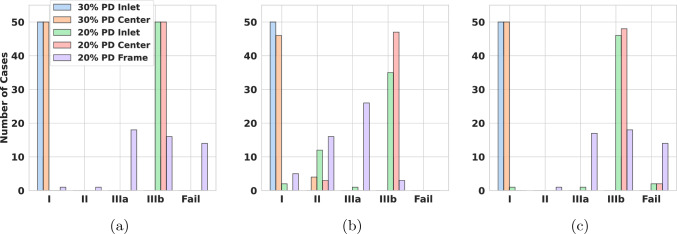
Classification of coil quality by RROC for 50 coils with different packing densities (PD) and at different catheter positions. **a** Small aneurysm geometry, **b** narrow neck aneurysm geometry, **c** bifurcation aneurysm geometry

### RROC

In this section, the RROC motivated occlusion classification from Sect. 3.3 is used to classify coil placements. We consider placements in two regions of the aneurysm. In a first test, the placements are generated according to the catheter positions as shown in Fig. 2. Secondly, we simulate placements in the center of mass of the aneurysms. Placements can vary by small perturbations, as it is the case in practice. This is implemented by adding a perturbation vector to the position of the catheter endpoint. The perturbations are sampled uniformly in a ball of radius 1 mm around the original catheter endpoint. In each aneurysm geometry and for each position, we simulate 50 of such placements and apply our modified occlusion classification. Figure 21 shows how placements are classified. We consider the PDs 20% and 30% while using filling coils for both PDs and a framing coil for 20% PD. Framing and filling coils distinguish in size and stiffness. For framing/filling coils we set $D_3$ to 5 mm/2 mm for the small aneurysm, 7 mm/4 mm for the narrow neck aneurysm and 8 mm/4 mm for the bifurcation aneurysm. The length $D_1$ is set to 50 $\upmu$m for framing coils and 40 $\upmu$m for filling coils, while $D_2$ is in all cases set to 305 $\upmu$m.

*Small Aneurysm* Fig. 21a: For the small aneurysm, packing densities of 30% lead in all cases to Class I. Class II is not assigned except for one of the framing coils. Class IIIa is roughly assigned to one third of the framing coils and Class IIIb to all the filling coils at 20% PD such as to one third of all the framing coils. The Class Fail is only assigned to the remaining framing coils.

We interpret the statistics as follows. Using filling coils with sufficient (30%) PD will result in good occlusion while this is not the case when lowering the PD of the filling coils to 20%. Framing coils on the other hand can in some cases produce at 20% the slightly better occlusion Class IIIa but might also produce failed cases where coil is migrating into the parent vessel.

*Narrow Neck Aneurysm * Fig. 21b: In case of the narrow neck aneurysm, all coils at PD 30% that were inserted close to the inlet resulted in Class I. For coils inserted at the center with the same PD a few are not in Class I. We also note that there is a small chance for coils of lower packing density to produce a Class I occlusion. Class II is mainly dominated by framing coils and coils that are inserted at the inlet having a lower PD. Note that also some high PD coils are assigned to this class. The majority of the framing coils is assigned to Class IIIa while the majority of the filling coils of 20% PD is assigned to Class IIIb. For the narrow neck aneurysm the Fail Class was never assigned.

*Bifurcation Aneurysm * Fig. 21c: For the bifurcation aneurysm, all cases with 30% PD are assigned to Class I. Class II is only assigned to one of the framing coils. Class IIIa mostly constitutes framing coils. The Class IIIb contains the majority of the lower PD filling coils and a third of the framing coils. Finally, the Class Fail includes another third of the framing coils and a small fraction of the low PD filling coils.

**Fig. 22:**
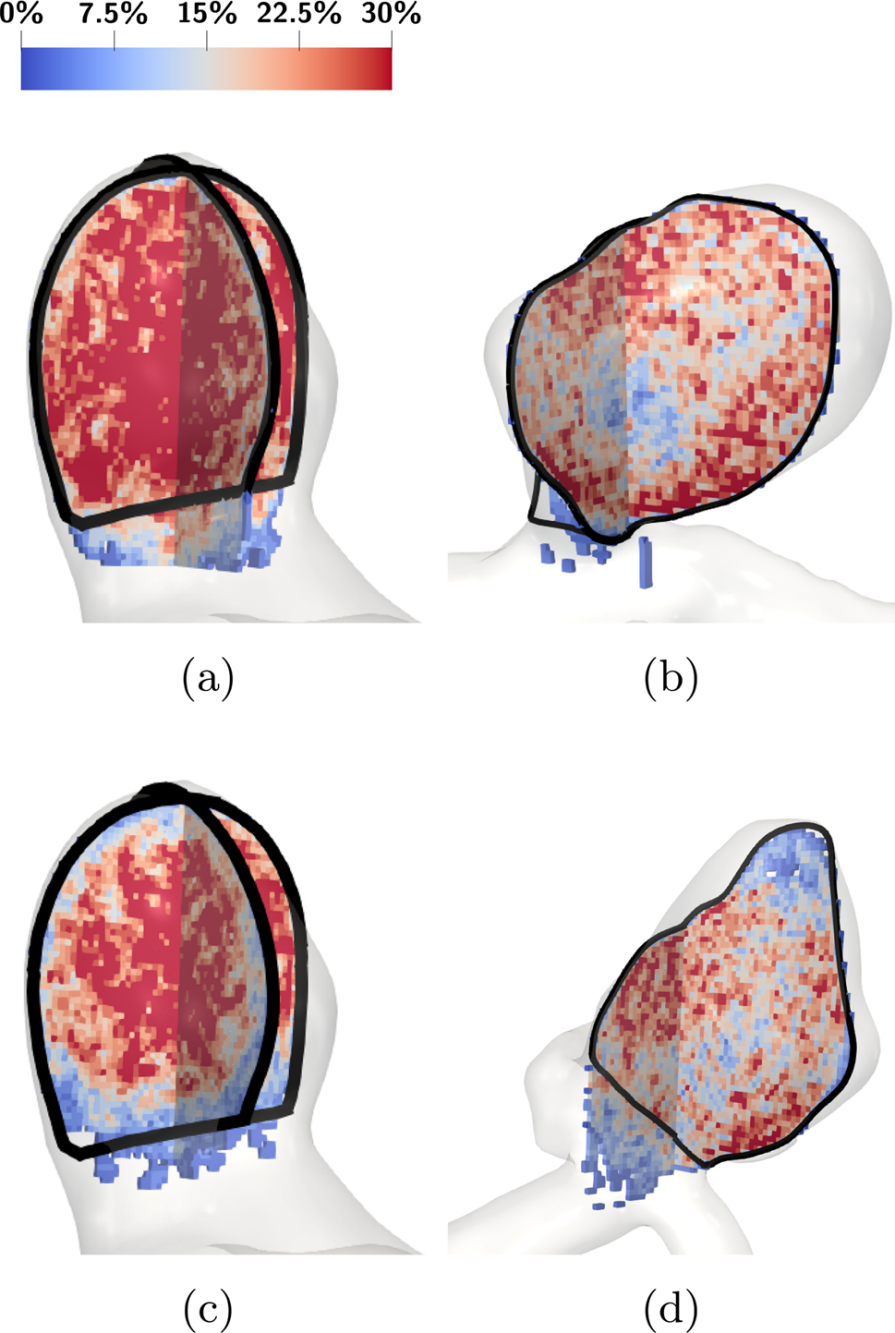
Examples of classes assessed by our classifier in Fig. 21 depicted as cross sections of averaged coil distributions with a sample size 50. The color scale corresponds to the PD in the voxels with values over 30% being red. **a** Class I from 30% PD Inlet 21a, **b** lies between Class II/IIIa from 20% PD Frame for the narrow neck aneurysm in Fig. 21b, **c** ClassIIIb from 20% PD Frame in 21a and **d** Class Fail from 20% PD Inlet in 21c

In Fig. 22, we show the averaged coil distributions of the 50 coils considered in each case calculated by (25). Figure 22a corresponds to Class I. Here the coil distribution is relatively uniform and sufficiently enough coil covers the neck without extensively wandering into the parent vessel. Figure 22b is a representative of Class II and IIIa. Some samples of this average get assigned to Class IIIa due to the low value of the coil distribution in the core. Another large fraction of samples gets assigned Class II since the coil distribution in the neck is not sufficient. Figure 22c shows a Class IIIb example. As in the RROC, the classification follows from the fact that the wall region at the neck of the aneurysm is poorly filled. Finally, in Fig. 22d, a relatively large amount of coil is located below the neck resulting in case fail classification.

## Discussion

In this section, we reflect on the numerical results of our coil distribution study as well as on the RROC-type classification.

### Coil distribution study

Our observations in Sect. 4.5 can be summarized in the following way.

*Influence of* $E_{{w}}$: Changing $E_{{w}}$ has a smaller impact than changing the other parameters. In contrast to Fujimura et al. (2018), increasing the stiffness $E_{{w}}$ of the helical coil reduced the presence of the coil within the boundary region. This is possibly due to the fact that the coil has a fixed diameter $D_3={4\,\mathrm{\text {m}\text {m}}}$. An increased Young’s modulus provides the coil with more structure, therefore moving away from the boundary where aneurysm and coil’s curvature do not match. This effect is prominent in the helical coil.

*Influence of* $D_2$: The influence of $D_2$ on the coil volume fractions is higher compared to the other parameters. A larger $D_2$ value leads to a more rapid filling of the aneurysm but decreases coil presence in the neck and boundary regions. Our study suggests that for a fixed coil length more coil is present in the aneurysm when increasing $D_2$, see also Babiker et al. (2013). In Kaesmacher et al. (2016), a comparison of large diameter coils and standard diameter ones showed that treatments with the latter ones lead to better RROC grades and less recanalization. They believe that large diameter coils rapidly fill the aneurysm while leaving the neck open. This is also confirmed by our simulation results. Better occlusion is then reached by subsequently treating the remaining neck region with standard coils.

*Influence of* $D_3$: For the helix coil, a higher $D_3$ causes a decrease in the coil presence within the neck area. This effect was experimentally observed in Ito et al. (2018), where the distance from the coil centroid position to the aneurysm neck increased for larger $D_3$. They explain that the increase in $D_3$ increases the coil loop sizes that expand in the aneurysm, ultimately pushing its centroid closer to the aneurysm center. This is consistent with our study, as a larger $D_3$ increased the coil concentration in the aneurysm boundary region while retaining a nearly constant volume in the aneurysm. Finally, our study confirms that larger $D_3$ values lead to a higher coil density within the boundary area of the aneurysm, which is commonly observed for those types of framing coils.

### RROC interpretation

The classification brings us to the following conclusions. In nearly all cases that were classified a packing density of ${20\mathrm{\%}}$ was not sufficient to be graded better than Class III. The reason is that we calibrated the classifier to give better occlusion grades for larger packing densities and when sufficient coil volume is present in the neck of the aneurysm.

**Small aneurysm geometry** Using filling coils with sufficient (30%) PD will result in good occlusion while this is not the case when lowering the PD of the filling coils to 20%. Framing coils on the other hand can in some cases produce at 20% the slightly better occlusion Class IIIa but might also produce failed cases where coil is migrating into the parent vessel.

**Narrow neck aneurysm geometry** The narrow neck aneurysm is occluded better when compared to the small aneurysm since fewer cases are assigned to Class IIIb and the Class Fail. On the other hand, the narrow neck is more challenging to fill which we see in the increased amount of coils assigned to Class II.

**Bifurcation aneurysm geometry** The bifurcation aneurysm behaves similar to the small aneurysm in terms of RROC. It is interesting to see that even though the neck diameter is considerably larger for this aneurysm, some framing coils can still occlude the aneurysm neck to give Class IIIa. This suggests medium- and large-necked aneurysms behave similarly when a framing coil is placed.

Our RROC motivated classification provides an extension to the traditional RROC and is based on the geometric distribution of the coil, therefore it can be used to speed up classification analyses of coil occlusion when correctly calibrated, for example, by means of subsequent hemodynamic flow simulations and analyses in virtually coiled aneurysms, more directly corresponding to the original RROC. This is planned to be part of future research articles.

## Conclusion

We have presented a mathematical model of endovascular coiling that is based on the DER method. Our model accurately takes into account important properties of the coil design such as its natural shape, bending and torsion response and can be placed efficiently into realistic aneurysm geometries. The broad applicability of the model was shown by testing it for the coiling of three different aneurysms showing that it is applicable in a wide range of cases. We have qualitatively validated the correctness of our model by comparing it to real coils extruded into free space.

The second part of our study focused on a statistical evaluation of our model. The main tool to achieve this was the voxelization of the coil geometry, which allowed us to define the notion of averaged coils. These were then used to calculate the volume fractions of coils in the core, boundary and neck region of an aneurysm and to study the parameter sensitivity of our coils. Further we have developed a Raymond–Roy occlusion-type classifier that allowed us to grade embolized coils by their occlusion properties. Finally, the relevance of the assigned classes were illustrated by analyzing mean fields of embolized coils for which a majority was graded with a specific class.

## Data Availability

The datasets generated during the current study are available upon request from the corresponding author.

## Appendix A Derivation of the twisting momentum

The material frame is parametrized by the Bishop frame as:

$$\begin{aligned} {\varvec{D}}_1^j&= \cos (\phi ^j){\varvec{U}}^j + \sin (\phi ^j){\varvec{V}}^j\\ {\varvec{D}}_2^j&= -\sin (\phi ^j){\varvec{U}}^j + \cos (\phi ^j){\varvec{V}}^j. \end{aligned}$$

From that, it follows

$$\begin{aligned} \frac{\partial {\varvec{D}}_1^j}{\partial \phi ^j} = {\varvec{D}}_2^j,\quad \frac{\partial {\varvec{D}}_2^j}{\partial \phi ^j} = -{\varvec{D}}^j_1. \end{aligned}$$

Now, we recall the form of the bending energy

$$\begin{aligned} E_{b1}&=\sum \limits _{i=1}^{n-2} \frac{b}{2{\overline{\ell }}}(\kappa _{i1}-\overline{\kappa _{i1}})^2,\\&\text { with } \kappa _{i1} = \frac{1}{2} \big ({\varvec{D}}_2^{i-1} + {\varvec{D}}_2^{i}\big ) \cdot \underbrace{\frac{2 {\varvec{t}}^{i-1}\times {\varvec{t}}^{i}}{1+{\varvec{t}}^{i-1}\cdot {\varvec{t}}^{i}}}_{(\kappa {\varvec{b}})_i},\\ E_{b2}&=\sum \limits _{i=1}^{n-2} \frac{b}{2{\overline{\ell }}}(\kappa _{i2}-\overline{\kappa _{i2}})^2, \\&\text { with } \kappa _{i2} = -\frac{1}{2} \big ({\varvec{D}}_1^{i-1} + {\varvec{D}}_1^{i}\big ) \cdot \underbrace{\frac{2 {\varvec{t}}^{i-1}\times {\varvec{t}}^{i}}{1+{\varvec{t}}^{i-1}\cdot {\varvec{t}}^{i}}}_{(\kappa {\varvec{b}})_i}. \end{aligned}$$

Let $\alpha :=b/(2{\overline{\ell }})$ and recall ${\varvec{t}}^{i}=\frac{{\varvec{x}}^{i+1}-{\varvec{x}}^{i}}{\Vert {\varvec{x}}^{i+1}-{\varvec{x}}^{i}\Vert }$. We proceed by calculating the gradient. First note that:

$$\begin{aligned} \frac{\partial \kappa _{i1}}{\partial \phi ^ i}&= \frac{1}{2} \frac{\partial {\varvec{D}}_2^i}{\partial \phi ^ i}\cdot (\kappa {\varvec{b}})_i =-\frac{1}{2} {\varvec{D}}_1^i\cdot (\kappa {\varvec{b}})_i \text { and } \\ \frac{\partial \kappa _{i+1,1}}{\partial \phi ^ i}&= \frac{1}{2} \frac{\partial {\varvec{D}}_2^i}{\partial \phi ^ i}\cdot (\kappa {\varvec{b}})_{i+1} =-\frac{1}{2} {\varvec{D}}_1^i\cdot (\kappa {\varvec{b}})_{i+1},\\ \frac{\partial \kappa _{i2}}{\partial \phi ^ i}&= -\frac{1}{2} \frac{\partial {\varvec{D}}_1^i}{\partial \phi ^ i}\cdot (\kappa {\varvec{b}})_i =-\frac{1}{2} {\varvec{D}}_2^i\cdot (\kappa {\varvec{b}})_i \text { and } \\ \frac{\partial \kappa _{i+1,2}}{\partial \phi ^ i}&= -\frac{1}{2} \frac{\partial {\varvec{D}}_1^i}{\partial \phi ^ i}\cdot (\kappa {\varvec{b}})_{i+1} =-\frac{1}{2} {\varvec{D}}_2^i\cdot (\kappa {\varvec{b}})_{i+1}. \end{aligned}$$

Using this for the gradient, it yields:

$$\begin{aligned} \frac{\partial E_{b1}}{\partial \phi ^ i} =&\alpha \frac{\partial }{\partial \phi ^ i} \big [ (\kappa _{i1}-\overline{\kappa _{i1}})^2 + (\kappa _{i+1,1}-\overline{\kappa _{i+1,1}})^2\big ] \\ =&-\alpha \big [ (\kappa _{i1}-\overline{\kappa _{i1}}) {\varvec{D}}_1^i\cdot (\kappa {\varvec{b}})_i \\&+ (\kappa _{i+1,1}-\overline{\kappa _{i+1,1}}) {\varvec{D}}_1^i\cdot (\kappa {\varvec{b}})_{i+1} \big ],\\ \frac{\partial E_{b2}}{\partial \phi ^ i} =&\alpha \frac{\partial }{\partial \phi ^ i} \big [ (\kappa _{i2}-\overline{\kappa _{i2}})^2 + (\kappa _{i+1,2}-\overline{\kappa _{i+1,2}})^2\big ] \\ =&-\alpha \big [ (\kappa _{i2}-\overline{\kappa _{i2}}) {\varvec{D}}_2^i\cdot (\kappa {\varvec{b}})_i \\&+ (\kappa _{i+1,2}-\overline{\kappa _{i+1,2}}) {\varvec{D}}_2^i\cdot (\kappa {\varvec{b}})_{i+1} \big ]. \end{aligned}$$

For the derivatives with respect to $\phi ^0$ and $\phi ^{n-2}$, we get

$$\begin{aligned} \frac{\partial E_{b1}}{\partial \phi ^ 0}&= \alpha \frac{\partial }{\partial \phi ^ 0} (\kappa _{1,1}-\overline{\kappa _{1,1}})^2 \\&= -\alpha (\kappa _{1,1}-\overline{\kappa _{1,1}}) {\varvec{D}}_1^0\cdot (\kappa {\varvec{b}})_{1},\\ \frac{\partial E_{b2}}{\partial \phi ^ 0}&= \alpha \frac{\partial }{\partial \phi ^ 0} (\kappa _{1,2}-\overline{\kappa _{1,2}})^2 \\&= -\alpha (\kappa _{1,2}-\overline{\kappa _{1,2}}) {\varvec{D}}_2^0\cdot (\kappa {\varvec{b}})_{1},\\ \frac{\partial E_{b1}}{\partial \phi ^ {n-2}}&= \alpha \frac{\partial }{\partial \phi ^ {n-2}} (\kappa _{n-2,1}-\overline{\kappa _{n-2,1}})^2 \\&= -\alpha (\kappa _{n-2,1}-\overline{\kappa _{n-2,1}}) {\varvec{D}}_1^{n-2}\cdot (\kappa {\varvec{b}})_{n-2},\\ \frac{\partial E_{b2}}{\partial \phi ^ {n-2}}&= \alpha \frac{\partial }{\partial \phi ^ {n-2}} (\kappa _{n-2,2}-\overline{\kappa _{n-2,2}})^2 \\&= -\alpha (\kappa _{n-2,2}-\overline{\kappa _{n-2,2}}) {\varvec{D}}_2^{n-2}\cdot (\kappa {\varvec{b}})_{n-2}. \end{aligned}$$

## Appendix B Oil simulation parameters

**General parameters**

We first state in Table 3 the material parameters that were used in each of the conducted simulations if not stated otherwise.

**Table 3:** Summary of simulation and material parameters

Material parameter	Value, Unit
$E_{{w}}$	230 GPa
$\mu _{{w}}$	0.4
$\rho$	21 $\times 10^{3} \textrm{kg m}^{-3}$

Simulation parameter	Value, Unit
$\Vert {\varvec{v}}_{\textrm{push}}\Vert$	3 cm s$^{-1}$
$k_{{w}}$	4 $\times 10^{2} {\textrm{N m}}^{-1}$
$\gamma _{{w}}$	0.01 N s m$^{-1}$
$v_{\epsilon }$	1 $\times 10^{-8} {\textrm{m s}}^{-1}$
${\overline{\ell }}$	$D_2$
$\alpha$	1 $\times 10^{-1} {\textrm{J m}}^{-1}$
$\eta _{{\varvec{X}}}$	1 $\times 10^{-2} {\textrm{N s m}}^{-1}$
$\eta _{\varvec{\Phi }}$	1 Nms
$\eta _{\mu _{\textrm{slip,CW}}}$	0.6
$\eta _{\mu _{\textrm{stick,CW}}}$	0.9
$\eta _{\mu _{\textrm{slip,CC}}}$	0.6

The material parameters are chosen to match those of a platinum coil. The insertion velocity, $\Vert {\varvec{v}}_{\textrm{push}}\Vert$, is based on discussions with our medical partners. Since the simulation assumes rigid walls, $k_{{w}}$ and $\gamma _{{w}}$ were set such that no segments pass through walls. The value of $v_\varepsilon$ is set as in Gazzola et al. (2018) and $\alpha$ is chosen such that the inextensibility constraint is sufficiently satisfied, see again Sect. 4.1. The initial node distance ${\overline{\ell }}$ is set to the coil diameter $D_2$. Unlike in Babiker et al. (2013), no issues in the contact detection occurred when ${\overline{\ell }}<1.5\,D_2$. The damping $\eta _{{\varvec{X}}}$ is set to provide numerical stability and to model the drag force of the surrounding blood on the coil. For blood, we choose it to be 100 times higher that in the validation experiment where air is surrounding the coil. $\eta _{\varvec{\Phi }}$ is chosen empirically such that the natural shape of the coil can be recovered. Finally, the coefficients of friction are chosen higher than in the literature (Otani et al. 2020) to account for the fact that aneurysm walls are deformable. This deformation provides additional stability to the coil, which we aim to consider by the higher coefficients of friction (see explanation on contact algorithm in Sect. 1).

## Appendix C Axial strain energy

The axial strain in the continuous case is given by the difference of the axial strain in the current and reference configuration:

C1 $$\begin{aligned} \epsilon = {\varvec{x}}'(s) - \overline{{\varvec{x}}}'(s). \end{aligned}$$

In the discrete setting, we assume that the axial strain is constant on an edge ${\varvec{e}}^j$ resulting in

C2 $$\begin{aligned} \epsilon \approx \frac{\Vert {\varvec{e}}^j\Vert }{\Vert \overline{{\varvec{e}}}^j\Vert } - \frac{\Vert \overline{{\varvec{e}}}^j\Vert }{\Vert \overline{{\varvec{e}}}^j\Vert }. \end{aligned}$$

This allows to write the axial strain energy for the discrete setting as

C3 $$\begin{aligned} \int _0^L \alpha \epsilon ^2 {\text{d}}s&= \sum \limits _{j=0}^{n-2}\int _ {{\varvec{x}}_j}^{{\varvec{x}}_{j+1}}\alpha \epsilon ^2 {\text{d}}s \end{aligned}$$

C4 $$\begin{aligned}&\approx \sum \limits _{j=0}^{n-2} \frac{1}{2}\alpha \left(\frac{\Vert {\varvec{e}}^j\Vert }{\Vert \overline{{\varvec{e}}}^j\Vert } - 1\right)^2 \Vert \overline{{\varvec{e}}}^j\Vert , \end{aligned}$$

which is what we have stated above.

## Footnotes

1 https://www.blender.org/.

2 https://www.meshlab.net/.

3 Detailed information about the experiment, including coil number and manufacturer, is available upon request.

## Code availability

The code repository and simulation details, including the experimental setup, are available upon request from the corresponding author.
